# Endovascular approaches to neural interfacing: lessons from cardiovascular technologies

**DOI:** 10.3389/fnins.2026.1933040

**Published:** 2026-09-23

**Authors:** Leire Landeira-Suquia, Aaron Lee, Estelle A. Cuttaz, Josef Goding, Sam E. John, Rylie A. Green

**Affiliations:** 1 Department of Bioengineering, Imperial College London, London, United Kingdom; 2 Department of Biomedical Engineering, The University of Melbourne, Parkville, VIC, Australia

**Keywords:** bioelectronics, endovascular electronics, endovascular neural interfaces, neural interfaces, stent-electrode array

## Abstract

Endovascular neural interfaces (ENIs) have emerged as a minimally invasive alternative to traditional neural interfaces. These devices are implanted within blood vessels adjacent to neural tissue to record or stimulate neural activity across the vessel wall. This enables minimally invasive implantation through angiographic procedures and reduces the risk of neural tissue damage. As they are deployed in the intravascular space, ENIs have a similar technological evolution to cardiovascular devices and share many features and constraints. First-generation ENIs borrowed guide wires and catheters employed in cardiovascular procedures for endovascular neural recording but were limited to acute settings. Concurrently, the cardiovascular field explored the possibility of integrating electronics onto endovascular devices. Catheters have been used for endovascular stimulation as a treatment for cardiovascular conditions. Cardiovascular stents have been integrated with wireless sensors, enabling self-reporting stents to monitor implant health. In 2016, a breakthrough in the field of ENIs was achieved by leveraging the chronic stability of stents to support an electrode array. Exemplified by Synchron’s Stentrode™, stent electrode arrays have been successfully employed as a brain-computer interface for chronic motor cortex recording. Subsequent research in the field has focused on expanding applications and improving spatial resolution. Incorporating valuable lessons from the field of interventional cardiology will be key to accelerate ENI development, especially in the areas of device miniaturisation, enhancement of stimulation capabilities, and wireless communication. Addressing these limitations could revolutionise the treatment of neurological disorders, enabling high spatial resolution recording and stimulation possible through minimally invasive, accessible procedures.

## 1 Introduction

Endovascular neural interfaces (ENIs) have recently emerged as a promising platform for the diagnosis and treatment of a range of neurological disorders. Unlike traditional electrode arrays which are implanted in direct contact with neural tissue through open surgery, ENIs utilise catheter-based procedures for minimally invasive implantation within blood vessels (Sefcik et al., 2016). The proximity of the nervous system to vasculature that can be catheterised enables recording or stimulation of deep and diverse targets across the vessel wall (Teplitzky et al., 2014; Neudorfer et al., 2020; Thielen et al., 2023). As a result, this approach bypasses the known trade-off between invasiveness and spatiotemporal resolution that conventional neural interfaces (NIs) often face. Endovascular bioelectronics have a history of use within interventional cardiology, with applications broadening into autonomic nervous system modulation and brain computer interfaces (Ponikowski et al., 2011; Naksuk et al., 2016; Oxley et al., 2016; Fan et al., 2020). Integrating insights from the cardiovascular front will be key to maximising the potential of endovascular approaches for neural interfacing.

Traditional approaches to neural interfacing span a spectrum of invasiveness and capacity for resolving neural activity. Non-invasive NIs such as electrodes for scalp electroencephalography (EEG) or wearable electrodes interface with neural tissue without penetrating the skin. Signals recorded arise from large, distant neuron populations with limited spatial resolution and information density (Caiado and Ukolov, 2025). Devices implanted through open surgery in contact with neural tissue such as electrocorticographic arrays (ECoG) or peripheral nerve cuffs can enable local field potential recording with the possibility of interfacing with single neurons (Larson and Meng, 2020; Moon et al., 2024). However, communication is limited to neural populations near the surface that devices are placed on. Penetrating electrodes can interface with deeper cortical layers, achieving single cell resolution with minimal off-target effects (Luan et al., 2023). Implantation of devices in direct contact with neural tissue triggers a foreign body response that leads to scarring and inflammation (Campbell and Wu, 2018). This deteriorates signal quality by increasing impedance and decreasing signal-to-noise ratio (SNR). ENIs bypass this limitation by being deployed in the intravascular space, which grants them two key benefits. Firstly, the vascular system is deeply interconnected with the nervous system. Blood vessels with a diameter greater than 1 mm traverse within 1 cm of deep brain regions (fornix, nucleus accumbens, subthalamic nucleus), cerebral cortex (motor cortex) and spinal cord (Neudorfer et al., 2020; Opie and O’Brien, 2022; Hasan and Arain, 2023). Correspondingly, in the periphery nerves commonly run alongside vasculature in the neurovascular bundle. Thus, ENIs can be placed in close proximity to the target tissue without being in direct contact, minimising the risk of neural tissue damage (Oxley et al., 2016). Secondly, implantation can be performed through a minimally invasive angiographic procedure. By only requiring a small incision to catheterise vasculature and route the device to the target, this well-established approach avoids open surgery, reducing procedural risks (Keegan and Hicks, 2022; Omeh and Shlofmitz, 2023). Early work in ENIs mostly involved using guide wires and catheters typically employed in cardiovascular interventions for acute endovascular EEG recording (Penn et al., 1973; Nakase et al., 1995; Stoeter et al., 1995). The landmark development of the Stentrode™ by Synchron in 2016 enabled chronic, stable endovascular neural recording for the first time owing to the integration of electrodes on a cardiovascular stent, introducing the form factor of stent-electrode arrays (SEAs) (Oxley et al., 2016). The promising results achieved with the Stentrode™ have sparked a growing interest into ENIs to fully exploit their capabilities. However, despite their advantages, ENIs also face unique challenges compared to conventional implantable NIs that arise from being deployed within the vascular system. In this aspect, ENIs are inherently similar to many cardiovascular devices, regarding both their form factor and interactions with tissues. Considerations such as radial stiffness, thrombogenicity, haemocompatibility and the ability to maintain vessel patency dictate the suitability of ENIs. Vessel wall interactions are particularly important for stent-like form factors as persistent pressure can disrupt wall function. Understanding the composition of blood vessel walls is key to the design of endovascular technologies.

Blood vessels are comprised of three concentric layers: the tunica intima, tunica media and tunica adventitia (Tucker et al., 2023). The innermost layer (tunica intima) consists of endothelial cells that provide an anti-thrombogenic and frictionless interface with blood (Félétou, 2011). The intermediate layer (tunica media) is primarily composed of smooth muscle cells and elastic fibres that regulate vessel diameter and vascular tone (van Thiel et al., 2017). The outermost layer (tunica adventitia) is mostly comprised of connective tissue that provides structural support to the vessel (van Thiel et al., 2017). The thickness of each of these layers varies across different blood vessels depending on their function. Arteries and arterioles have a thick tunica media layer which is reduced in the central nervous system (CNS) (Tucker et al., 2023). Conversely, veins have a very thin tunica media as well as valves to ensure unidirectional flow (Tucker et al., 2023). Moving from the heart towards peripheral tissues, blood vessels decrease in diameter and wall thickness (Tucker et al., 2023). In the context of ENIs, this means that placement within smaller vessels decreases the electrode-target distance with implications for reduced stimulation thresholds and increased spatial resolution. At the same time, smaller vessels are more prone to piercing and call for smaller devices to maintain vessel patency.

The interface between the device and the endothelium is crucial to promote tissue integration. Damage to endothelial cells triggers a healing response dependent on the degree of injury. Denudation of the endothelium results in endothelialisation, which is the proliferation and migration of endothelial cells to cover the device (Marei et al., 2022). This restores the natural anti-coagulant, non-thrombogenic interface between blood and the vessel wall. However, if the injury exposes the subendothelial matrix, it can trigger the coagulation cascade and lead to thrombogenesis (Marei et al., 2022). Additionally, chronic mechanical stress on the vessel wall can result in excessive proliferation of smooth muscle cells, resulting in lumen reduction or neointimal hyperplasia (Cornelissen and Vogt, 2018). Consequently, the mechanical properties of the materials employed are important, since stiff materials will cause additional damage to vessel walls. Material haemocompatibility is also key to prevent thrombogenesis, haemolysis, and inflammation (Kuchinka et al., 2021). Devices must be optimised to cause minimal disruption to normal haemodynamics (John et al., 2022). For this, profile geometry and conformability are critical design considerations.

Prior reviews on ENIs focus on advancements within the neural engineering field (Fan et al., 2020; Soldozy et al., 2020; Thielen et al., 2023; Liu et al., 2025; Ognard et al., 2025). Yet, there remains a valuable opportunity to integrate extensive learnings from the cardiovascular field for further ENI development. Whilst comparisons have been drawn between ENIs and pacemakers with respect to connection lead safety, broader considerations such as device design, fabrication techniques and the implication of endovascular stimulation remain largely unexplored (Raza et al., 2020). Since the development of cardiac catheterisation techniques in 1929, cardiovascular interventions have progressively shifted towards endovascular approaches (Mueller and Sanborn, 1995). The electroactive nature of cardiac tissue lends itself to the integration of electronics. This same principle also applies to neural tissue, which has allowed repurposing of devices for neural applications (Penn et al., 1973). Figure 1 shows a timeline highlighting some key developments in endovascular technologies, focusing on neural applications in blue and cardiovascular applications in red. The latter specifically features milestones that are tightly connected to the development of ENIs, with a focus on SEAs. Leveraging the existing knowledge in the field of interventional cardiology will be key to the rapid ENI development while advancement of ENIs, in turn, could benefit cardiovascular applications. To this end, this review explores advancements on endovascular electronics for both cardiac and neural applications, bridging the gap between these two fields to accelerate their development and create avenues for future opportunities.

**Figure 1:**
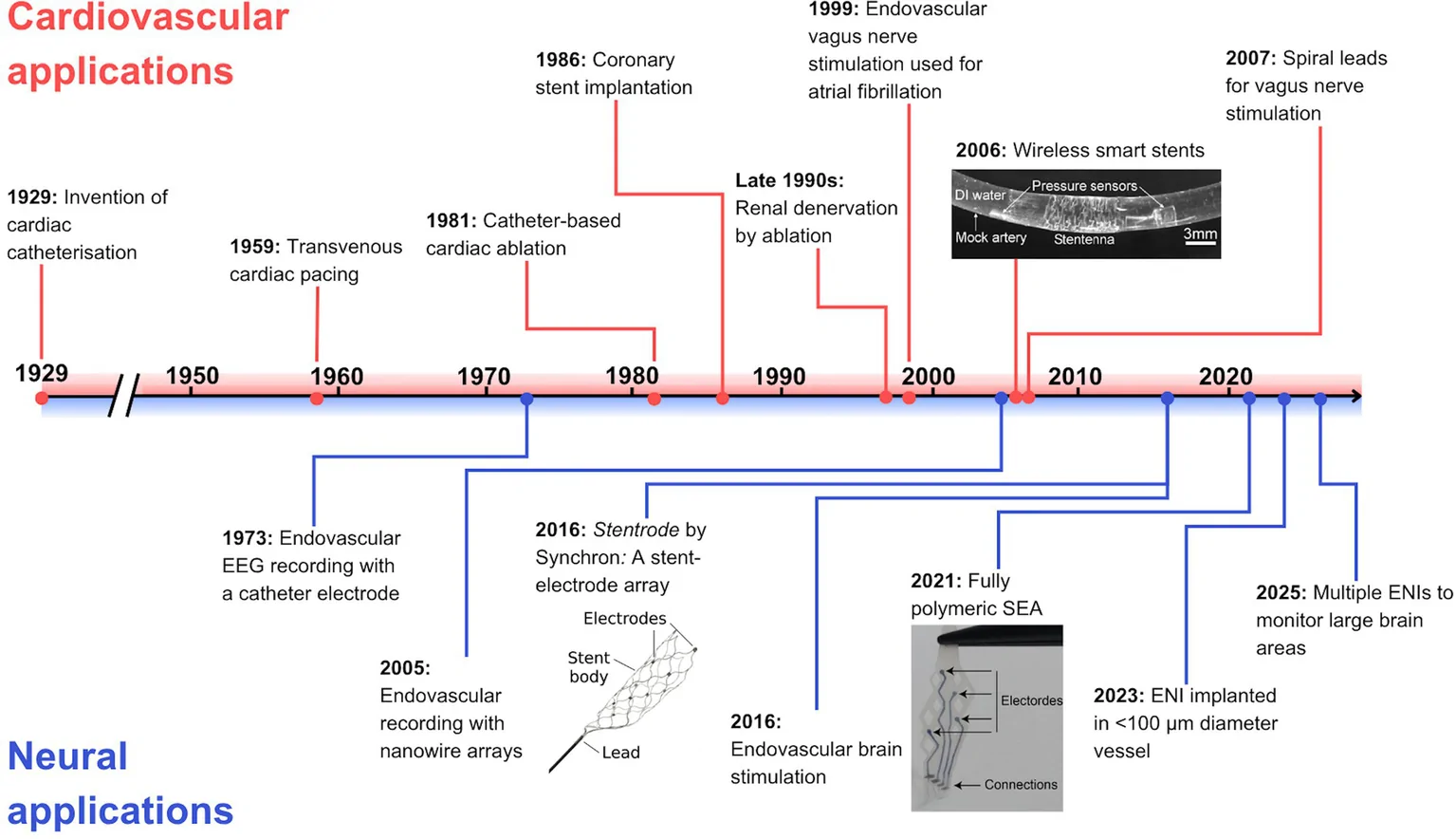
Timeline of key developments in endovascular bioelectronics for cardiovascular (red) and neural (blue) applications (Furman and Schwedel, 1959; Penn et al., 1973; Scheinman, 1986; Mueller and Sanborn, 1995; Schauerte et al., 1999; Llinás et al., 2005; Takahata et al., 2006; Nabutovsky et al., 2007; Roguin, 2011; Gaba et al., 2016; Oxley et al., 2016; Fanelli et al., 2022; Zhang et al., 2023; Masuda et al., 2025; Mulè et al., 2026). Chronologically, figures adapted with permission from Takahata et al. (2006), Copyright © 2006 IEEE; Chetty et al. (2025) used under CC BY 4.0; Fanelli et al. (2022) used under CC BY 4.0. Figure produced using Canva.

## 2 Endovascular electronics in cardiac applications

Endovascular technologies for neural recording and stimulation draw heavily from the principles developed for catheter-based procedures used in interventional cardiology. The unique access provided by endovascular approaches created an opportunity for integrating electronics into catheters and stents. Catheters equipped with electrodes provided early demonstration of endovascular stimulation of nearby targets outside vessels (Schauerte et al., 1999; Mulè et al., 2026). Cardiovascular stents arise from the necessity for chronic devices seamlessly integrating with the vascular system. Further developments include the integration of sensing and wireless communication (Takahata et al., 2006; Shedden et al., 2010).

### 2.1 Catheter-based endovascular stimulation

Catheterisation techniques have paved the way for endovascular stimulation as exemplified by the use of transvenous leads for cardiac pacing (Furman and Schwedel, 1959). Wire geometries with an exposed electrode tip have allowed electrical stimulation to different anatomical targets via vascular catheters (Table 1). Early work showed that high frequency currents could be delivered to selectively ablate tissue as a treatment for arrhythmias, which soon expanded to nearby nerves (Scheinman, 1986; Mulè et al., 2026). The renal and vagus nerves were the first targets of endovascular nerve stimulation due to their proximity to catheter-navigable vessels and their critical role in controlling cardiovascular function (Schauerte et al., 1999; Madhavan et al., 2014).

**Table 1:** Collection of the key parameters and characteristics of studies on catheter-based endovascular peripheral nerve stimulation for the treatment of cardiovascular stimulation (N.R., not reported).

Citation	Implantation vessel (subject / model)	Implantation duration	Electrode number	Electrode size	Form factor	Safety outcomes	Stimulation parameters	Performance metrics
Madhavan et al. (2014)	Renal vein (dog, baboon)	Acute	4	N.R.	Straight catheter	Hemorrhage (1 of *n* = 7)	10 V	13.0 ± 3.3 mmHg and 10.2 ± 4.6 mmHg increase in systolic and diastolic blood pressure
Naksuk et al. (2016)	Unilateral or bilateral renal vessel, hepatic vein, hepatic artery, portal vein, celiac artery (dog)	Acute	6 – 8	N.R.	Spiral catheter	Skeletal muscle contraction, fasciculations	1 – 150 V	5 – 7 mmHg average increase in mean arterial pressure
Schauerte et al. (1999)	Superior vena cava, right pulmonary artery (dog)	Acute (10 h)	N.R.	N.R.	Basket catheter	No complications reported	28 V	50% decrease in ventricular contraction frequency
Schauerte et al. (2001)	Superior vena cava, coronary sinus (human)	Acute	8 or 10	N.R.	Straight catheter	Pain, chest discomfort, coughing	10 – 30 V	Heart rate slowing effects
Hasdemir et al. (2003)	Superior vena cava (dog)	Acute (4 h)	10	Length: 1 mm	Lasso catheter	No complications reported	15 ± 7 V	50% decrease in heart rate
Nabutovsky et al. (2007)	Superior vena cava (dog)	Acute	6	Length: 5 mm	Spiral catheter	No complications reported	7.5 V	21.4% decrease in heart rate
Ponikowski et al. (2011)	Right/left brachiocephalic veins, left pericardiophrenic vein (human)	Acute (overnight)	N.R.	N.R.	Straight catheter	Thrombus formation (1 of *n* = 16)	5.3 ± 2.1 mA (left) and 3.5 ± 1.6 mA (right)	90% reduction in central sleep apnea episodes

One of the earliest examples of transmural targeting of nerves from within a blood vessel is renal denervation. Renal denervation employs endovascular electrical stimulation to deliver radiofrequency energy and ablate nerve fibres (Weber et al., 2019). Stimulation is aimed at reducing renal sympathetic signalling to the kidneys due to their role as a blood pressure regulator. Although invasive surgical approaches to renal denervation had shown promise for blood pressure control, the high rates of perioperative morbidity prevented widespread adoption (Weber et al., 2019). Due to the proximity of renal nerves and the renal artery, endovascular denervation was presented as an alternative. This procedure was initially performed in a point-by-point manner, using a single electrode catheter (Figure 2A) and rotating it to generate multiple lesions in a helical pattern (Weber et al., 2019). Novel devices were subsequently developed to improve wall contact and decrease procedure time, including balloon catheters mounted with spiral electrodes (Figure 2B) as well as spiral leads with multiple electrode contacts (Verheye et al., 2015; Akrawinthawong and Yamada, 2024; Figure 2C). By using spiral electrode geometries, multiple sites along the renal nerve could be stimulated in a helical shape simultaneously from within the renal artery. For patients presenting with drug-resistant hypertension, targeted denervation through endovascular electrical stimulation offers substantial and persistent reduction in blood pressure (Krum et al., 2009).

**Figure 2:**
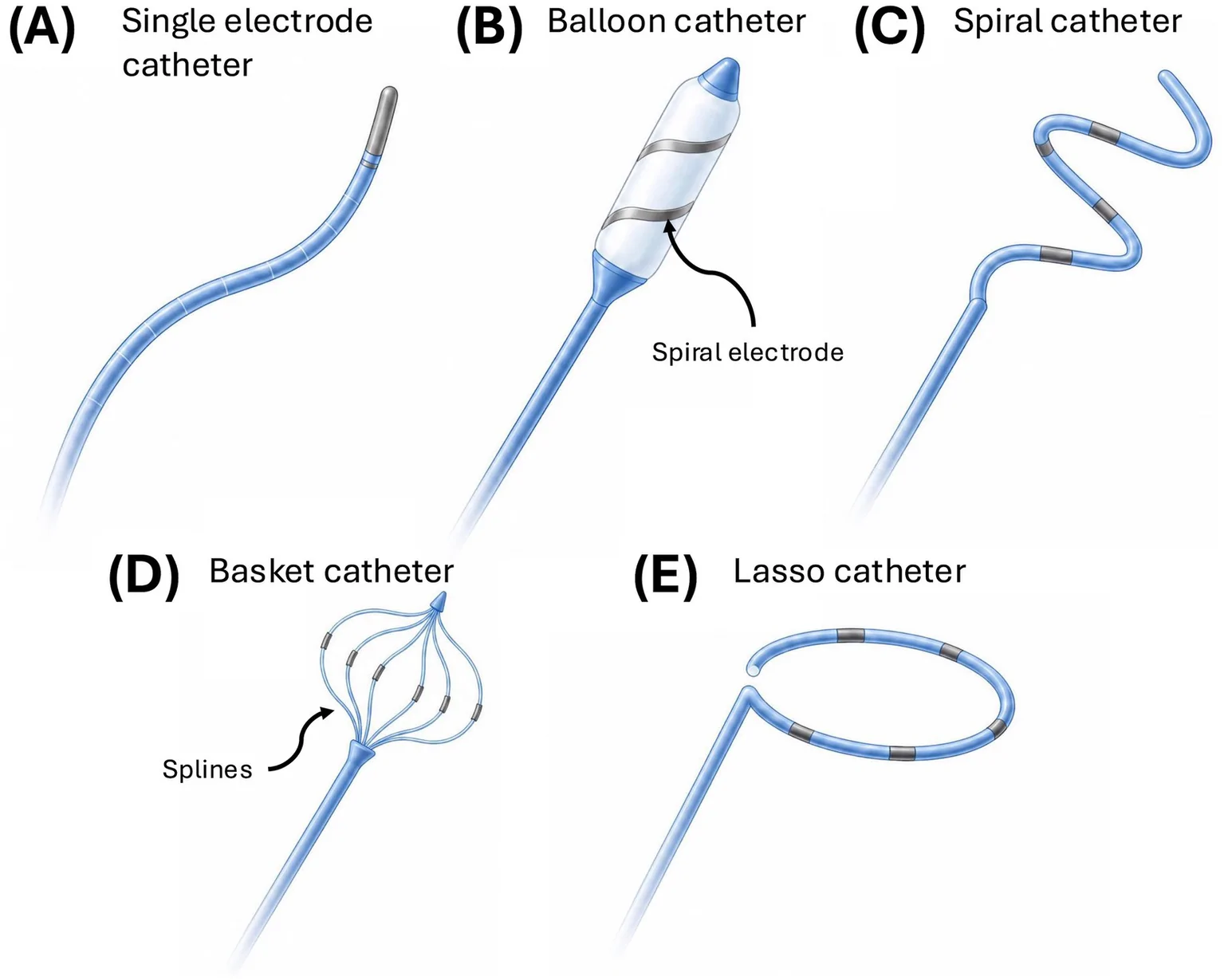
Schematic of different catheter designs. **(A)** Single electrode catheter. **(B)** Balloon catheter with a spiral electrode. **(C)** Spiral catheter with four electrodes. **(D)** Basket catheter with an electrode on each spline. **(E)** Lasso catheter with five electrodes. Figure generated using Illustrae.

The efficacy of renal denervation has led to researchers exploring the possibility of using renal nerve stimulation to increase blood pressure. One potential application is in neurocardiogenic syncope, a condition commonly described as “fainting” or “passing out” due to a hypotensive episode, typically resulting from the combination of bradycardia and vasodilation (Madhavan et al., 2014). As this condition lacks an effective therapy, endovascular stimulation of the autonomic nervous system could be employed to transiently raise blood pressure. High frequency stimulation (800–900 Hz) with an amplitude of 10 V delivered through a quadripolar catheter in the renal vein was shown to increase systolic blood pressure in canine models. Blood pressure returned to baseline upon cessation of stimulation, showing that effects are driven by continuous nerve firing and are compatible with physiological homeostatic processes. Optimal stimulation parameters and locations were further investigated by employing both a quadripolar catheter and multipolar spiral catheters implanted in a variety of blood vessels (Naksuk et al., 2016). Transient increases in blood pressure could be achieved from several positions along the vessel, with the optimal location remaining highly subject dependent. Stimulation amplitudes higher than 10 V were found to be needed for statistically significant blood pressure elevation, which also induced off-target activation resulting in abdominal contractions in 22% of cases. Reducing the activation thresholds will be key to minimise off-target effects and translate this approach into a viable clinical treatment.

Endovascular approaches have also been applied for neuromodulation of the parasympathetic nervous system. Primarily antagonistic to the sympathetic nervous system, parasympathetic signalling reduces heart rate and is primarily mediated by cardiac branches of the vagus nerve (Gordan et al., 2015). Stimulation could thus be employed to control increased ventricular rate. In 1999, a basket electrode catheter (Figure 2D) was found to achieve a sinus rate slowing effect when implanted in the superior vena cava or right pulmonary vein in dogs (Schauerte et al., 1999). The magnitude of the heart rate slowing effect was proportional to the amplitude of stimulation, ranging from a minor reduction to complete cardiac arrest. Heart rate returned to baseline upon cessation of stimulation. Stimulation amplitudes as high as 28 V were needed to achieve a 50 % decrease in ventricular contraction frequency. Similar effects were observed upon implantation of multipolar electrode catheters into the superior vena cava or coronary sinus of patients (Schauerte et al., 2001). Stimulation from the coronary sinus required lower voltage thresholds and resulted in fewer side effects. Despite this improvement, the use of high voltages was perceptible, and many patients reported chest pain or discomfort. While basket catheters may offer improved vessel wall contact over multipolar wire designs, they have also been suggested to carry a higher risk of thrombus formation between the splines (Hasdemir et al., 2003). To reduce thrombogenicity while retaining consistent and stable stimulation, an alternative ‘lasso catheter’ design was developed, consisting of a flexible loop with 10 electrodes (Figure 2E). By significantly reducing vessel obstruction, the lasso catheter could be safely deployed for extended time periods without thrombus formation and with decreased stimulation thresholds in comparison to a commercial basket catheter. Endovascular spiral vagal stimulators have subsequently been designed to maintain these benefits whilst following the anatomy of the target nerve. Different spiral diameters and pitches were compared to achieve close contact with the cardiac branches of the vagus nerve, which wreathe around the superior vena cava (Nabutovsky et al., 2007). Slightly increased radial force in the spiral catheter allows lower stimulation thresholds with comparable performance to basket catheter designs.

Another target for endovascular stimulation is the phrenic nerve. Conventional phrenic nerve stimulation through nerve cuff electrodes has been employed to regulate breathing patterns (Balasubramanian et al., 2025). Anatomically, the phrenic nerve lies adjacent to the right and left brachiocephalic veins and the left pericardiophrenic vein, which puts it in an ideal position for endovascular access (Stauffer et al., 2023). Endovascular stimulation of the phrenic nerve can help control respiration and has been explored as a treatment for central sleep apnoea in heart failure patients (Ponikowski et al., 2011). Stimulation leads were implanted for two nights in one of the adjacent veins depending on patient’s anatomy. It was found that the therapy reduced the number of apnoea episodes per night by 90 % using stimulation amplitudes less than 5 mA. This demonstrates that an endovascular implant for phrenic nerve stimulation could be employed as a chronic central sleep apnoea treatment, with potential for expansion to other indications such as cervical spinal cord injury. Despite a prominent cardiopulmonary focus, the use of endovascular approaches showed that both arteries and veins can be used for effective neuromodulation with clinically meaningful outcomes (Table 1) (Nabutovsky et al., 2007; Ponikowski et al., 2011; Naksuk et al., 2016; Weber et al., 2019). Across many of these applications, devices have been optimised to improve contact stability and reduce occlusion and thrombogenicity, while leveraging catheter placement for transluminal stimulation.

### 2.2 Smart stents for endovascular sensing

Cardiovascular stents are meshed tubular structures which are implanted to mechanically reopen narrowed or occluded blood vessels to restore blood flow (Scafa Udriște et al., 2021). As chronic devices, stents are designed to have a low profile and minimal footprint to conserve the normal blood-endothelium interface to the greatest possible extent. However, stenting can lead to inflammatory responses that trigger neointimal hyperplasia, characterised by the thickening of the vessel wall and loss of vessel patency (Cornelissen and Vogt, 2018). This process is known as in-stent restenosis, and is usually diagnosed once the patient becomes symptomatic, often a sign of severe artery blockage (Bateman et al., 2025). Active or self-reporting stents are embedded with sensors to enable real-time monitoring of implant health for earlier diagnosis (Vishnu and Manivasagam, 2020). By using the stent itself as an electrode, it was found that changes in impedance were correlated with the presence of tissue and could be used as an indicator of neointimal growth (Shedden et al., 2010). To improve sensitivity to changes in the vessel wall, continuous evaluation of stent health was explored using pressure sensors. By monitoring blood pressure and flow, these sensors indirectly measure stenosis or artery blockage. Capacitive pressure sensors integrated onto coil design geometries for the stent body allow for continuous monitoring with wireless communication, where the stent itself works as an antenna (Takahata et al., 2006; Figure 3A). In 2018, a similar device was shown to be capable of detecting blood clot formation in an *in vivo* swine model (Chen et al., 2018; Figure 3B).

**Figure 3:**
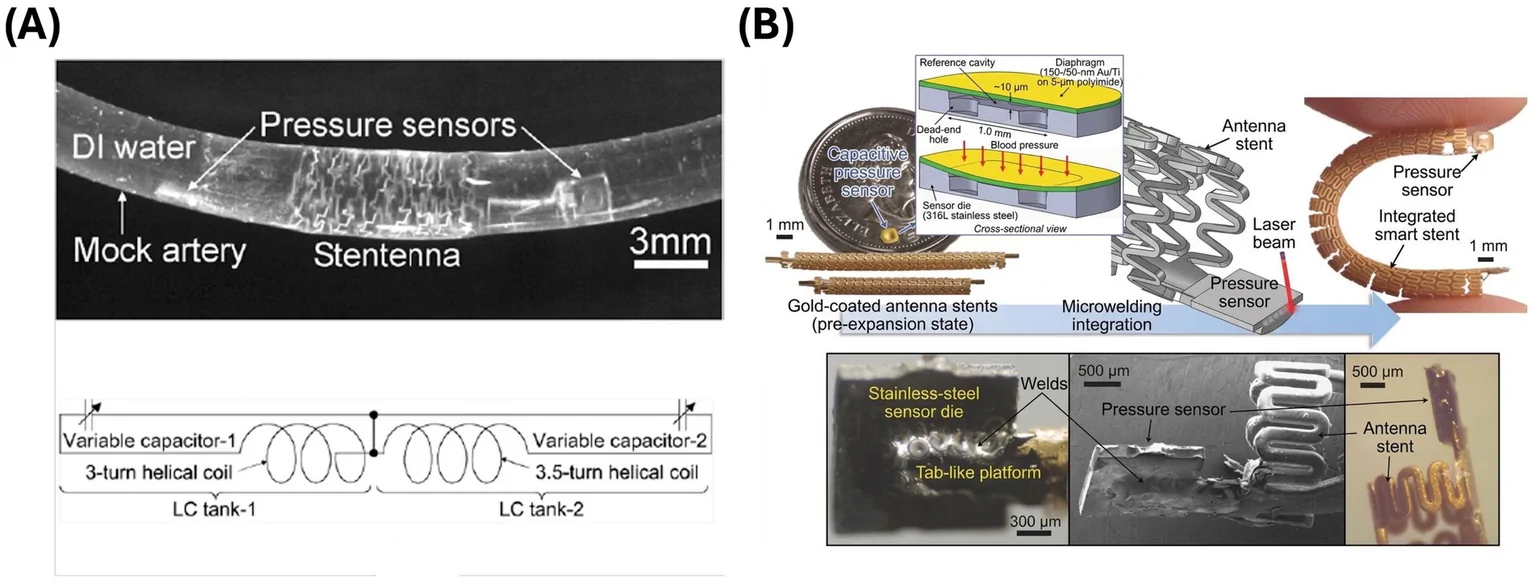
Wireless self-reporting stent devices. **(A)** Smart stent for monitoring intraluminal pressure and flow. Top: The deployed device in a mock artery. Bottom: Equivalent circuit of device after expansion. Adapted with permission from Takahata et al. (2006). Copyright © 2006 IEEE. **(B)** Smart stent to detect in-stent restenosis and occlusion. Top: From left to right, stents and pressure sensor, schematic of the sensor structure, schematic of sensor and stent welding process, and image of finalised device. Bottom: Close-up images of finalised device. Adapted from (Chen et al., 2018). Used under CC BY 4.0.

Incorporating wireless functionality into endovascular bioelectronics removes the need for wires to cross the vessel wall which mitigates the risk of infection and thrombogenesis (Xu et al., 2026). A key challenge of wireless bioelectronic implants is achieving safe and efficient power transfer at the required depths. A comparison between near-field wireless systems using inductive or capacitive coupling showed that both methods are suitable to safely power a stent at depths of up to 30 mm (Aldaoud et al., 2018). To further extend this working range and enable operation from any potential implantation site, long-range wireless electronic systems have been proposed instead (Bateman et al., 2025). A long-range system successfully diagnosed 50% stent-edge restenosis from up to 50 cm away in an *in vitro* set-up (Bateman et al., 2025). As seen in Figure 4A, the stent and pressure sensor were fabricated by laser cutting of a stainless-steel tube and copper film, respectively. Although the coil-like structure is beneficial to wireless communication, the lack of longitudinal links impacts the radial force of the stent. To address this, a hybrid metallic and polymeric stent was developed by incorporating polymeric links to a metal coil-shaped stent (Herbert et al., 2022). The stent was integrated with aerosol jet printed soft capacitive sensors of < 20 μm in thickness, highlighting the importance of low-profile sensors and devices to avoid disruption of haemodynamics. To enable precise placement of the sensors and their full integration with the stent, they could be incorporated directly during the stent fabrication process. For this, established photolithography techniques can be employed to fabricate the stent as a flat structure with a capacitive sensor in the centre (Oyunbaatar et al., 2023). Figure 4B shows the device, which was then rolled through a thermal treatment that results in residual surface stress and successfully employed for blood pressure and heart rate monitoring in a tissue phantom.

**Figure 4:**
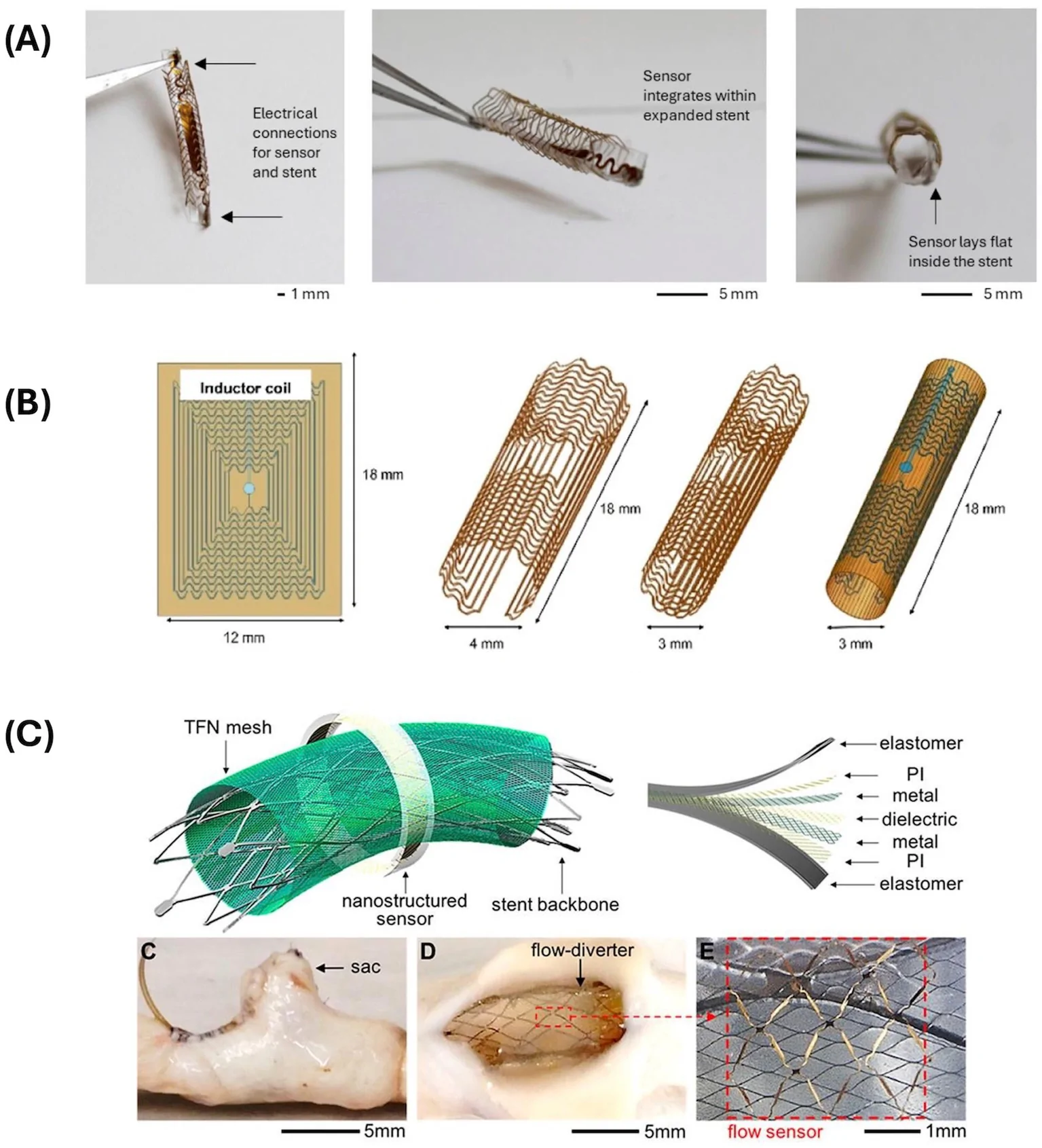
Self-reporting stent devices. **(A)** Images of fully assembled long-range wireless stent and pressure sensor system. Adapted from (Bateman et al. (2025). Used under CC BY 4.0. **(B)** Pressure sensor-integrated self-rollable stent. From left to right, 2D planar structure, rolling of inductor coil and 3D rolled structure of sensor-integrated stent mesh. Adapted with permission from Oyunbaatar et al. (2023). Copyright © 2023 American Chemical Society. **(C)** Flow diverter system for monitoring of intra-aneurysmal haemodynamics. Top: Schematic of flow diverter system with stent backbone, thin film nitinol mesh and nanostructured sensor. Multilayered pressure sensor structure. Bottom: Device implanted in pig aorta with aneurysmal sac and microscope image of the flow sensor. Adapted with permission from Howe et al. (2018). Copyright © 2018 American Chemical Society.

Continuous self-reporting on performance and status of endovascular devices can be used to diagnose and monitor in-stent restenosis and ensure that device performance meets minimal operational standards (Takahata et al., 2006; Chen et al., 2018; Oyunbaatar et al., 2023). The same principle has been used to integrate a cardiac pressure monitor on a stent to diagnose heart failure when implanted in the pulmonary artery (Chow et al., 2010). Additionally, a stent integrated with a thin film capacitive sensor and wired to a monitoring system was developed to detect pressure fluctuations in the superior sagittal sinus (SSS), which indirectly provides intracranial pressure measurements (Lee et al., 2025). Such a device could revolutionise intracranial pressure monitoring, which is currently limited to intensive care environments. A similar neurovascular smart stent seen in Figure 4C, has been developed to assess intra-aneurysmal haemodynamics for post-treatment monitoring, which is currently only possible through in-hospital imaging techniques (Howe et al., 2018). The device is a mesh-like flow sensor placed perpendicularly around a flow diverter stent. Changes in capacitance due to sensor deformation were used to quantify flow into the aneurysmal sac with high sensitivity demonstrated using an *in vitro* model. Following this work, similar approaches have been explored specifically for ENIs. A wireless power transfer system was recently developed to power a standard medical stent for endovascular ECoG (Xu et al., 2026). Near-field inductive coupling was employed to link an external device to a subcutaneous relay, which in turn uses capacitive coupling to deliver energy to unmodified stents placed in blood vessels. The system enables delivery of 45 mW, enough to continuously power recording from an ENI implanted in the SSS, which was the intended application. These studies show that stents provide a stable, reliable platform for the integration of electronic systems for a variety of applications (Table 2). This highlights the possibility of enhancing their functionalities beyond purely mechanical scaffolds and integrating sensing and wireless communication abilities. Although there has been a modest amount of *ex vivo* and *in vivo* acute animal studies, smart stents are yet to be translated to human trials.

**Table 2:** Collection of the key parameters and characteristics of studies developing smart stents (N.R., Not reported).

Citation	Application	Test platform	Stent fabrication	Active components	Performance metrics
Shedden et al. (2010)	In-stent restenosis	*Ex vivo* (swine)	Commercial stent	Impedance monitoring	Impedance changes associated with tissue growth
Takahata et al. (2006)	Wireless flow measurement	*In vitro*	Electrical discharge machining	Pressure sensors Wireless power transfer	Resonant frequency shift of 57.4 kHz/torr at a 1 cm distance
Chen et al. (2018)	In-stent restenosis	*In vivo* (swine)	Laser micromachining	Pressure sensors Wireless power transfer	Resolution of 12.4 mmHg in vitro 200 kHz frequency shift over 62 mmHg
Aldaoud et al. (2018)	Wireless endovascular system	*Ex vivo* (cow)	Metal wire coil	Wireless power transfer	Inductive: 1.9% efficiency at >30 mm depth Capacitive: 0.98% efficiency at 30 mm transmission depth
Bateman et al. (2025)	Stent edge restenosis	*In vitro*	Laser micromachining	Capacitive pressure sensors Wireless power transfer	10 MHz frequency shift for 100 mmHg change at > 50 cm transmission distance (air)
Herbert et al. (2022)	Monitoring blood pressure, flow, and pulse rate	*In vivo* (rabbit)	Laser micromachining	Pressure sensors Wireless power transfer	11 MHz frequency shift for 100 mmHg change at 2.5 cm transmission distance (air)
Oyunbaatar et al. (2023)	In-stent restenosis	*In vivo* (rat)	Photolithography	LC pressure sensors Wireless power transfer	0.1 MHz frequency shift for 100 mmHg change at 20 cm transmission distance (air)
Chow et al. (2010)	Cardiac monitoring	*In vivo* (swine)	Commercial stent	Capacitive pressure sensors Integrated circuit Wireless power transfer	Pressure resolution of 0.5 mmHg in regulated pressure chamber 42.2 kb/s channel data-rate
Lee et al. (2025)	Intracranial pressure monitoring	*In vivo* (swine)	Commercial stent	Pressure sensors	0.052 %/mmHg sensitivity
Howe et al. (2018)	Intra-aneurysmal haemodynamics monitoring	*In vitro*	Commercial stent	Capacitive flow sensors	0.032 m/s velocity change sensitivity
Xu et al. (2026)	Wireless endovascular neural recording	*Ex vivo* (cow)	Commercial stent	Wireless power transfer	45 mW power delivery

## 3 Endovascular electronics in neural interfacing

The success of angiographic delivery of cardiac devices has led to their repurposing for other applications. As with the use of endovascular approaches in cardiovascular applications, early feasibility work for neural recording and stimulation was performed with wire geometries. First generation ENIs mostly employed existing guidewires or catheters as recording electrodes, with the first instance of endovascular EEG recording dating back to 1973 (Penn et al., 1973). The success of stent-geometries for long-term vessel placement and integration of electronics was pivotal to the development of the Stentrode™ in 2016 for endovascular brain recording (Oxley et al., 2016). Subsequent work has focused on defining and pushing the boundaries of ENI technologies.

### 3.1 Guide-wire and catheter electrodes

Electrophysiological activity of deep brain regions cannot be readily detected using standard scalp EEG due to the physical distance from recording electrodes. Endovascularly deployed devices reduce this distance by being placed in deep neurovasculature adjacent to the source of the recorded signal (Table 3). In 1973, a magnetically guided catheter was adapted for use as an intravascular intracranial EEG recording device (Penn et al., 1973). Using the magnetic tip as a recording electrode, placement within the middle cerebral artery enabled detection of focal electrical activity, obtaining higher amplitude signals than through extracranial recordings. Similarly, Nakase et al. showed that intravascular neural recording could detect ictal activity in the left medial temporal lobe from within the middle cerebral artery with amplitudes up to five times greater than scalp EEG (Nakase et al., 1995). In 1997, Boniface and Antoun (Boniface and Antoun, 1997) used a similar stainless steel guidewire with a single platinum electrode implanted in the origin of the middle cerebral artery. The device was able to record epileptiform spikes in a patient suffering from intractable seizures, showing the potential of intravascular EEG to be used to localise epileptic foci. They also reported the persistence of pulse artifacts due to arterial placement, which deteriorated the signal. Guide wire approaches have been deployed through other vessels such as the middle meningeal artery, basilar artery and callosomarginal artery (Stoeter et al., 1995). While recording using an endovascular electrode, somatosensory evoked potentials and auditory evoked potentials could be detected from the middle meningeal and basilar artery, respectively. Depending on the vessel, brain region and type of activity, recorded neural signals could be between two to four times larger than scalp EEG or show negligible differences. Whilst most research thus far had focused on arteries, the veins and sinuses of the brain can also be used for navigation to deep brain targets for recording. In 1997, epileptiform activity was recorded from the temporal lobe to determine the ictal focus and spreading pattern, using a guide wire inserted into the cavernous sinus and superior petrosal sinus (Mikuni et al., 1997). Recordings were performed over an extended duration of 3 – 40 h, and the intravenous electrode detected discharges that were not present in simultaneous extracranial recordings. These early instances of ENIs consistently demonstrated that endovascular recordings achieve greater SNR than extracranial approaches.

**Table 3:** Collection of the key parameters and characteristics of studies on catheter-based ENIs (N.R., not reported).

Citation	Implantation vessel (subject / model)	Implantation duration	Electrode number	Electrode size	Form factor	Safety outcomes	Recording or stimulation	Performance metrics
Penn et al. (1973)	Middle cerebral artery (baboon, human)	Acute	1	Length: 1.5 mm Diameter: 0.6 mm	Guide wire	No complications reported	Recording	Impedance: 70 – 100 Ω Superior performance to scalp EEG
Nakase et al. (1995)	Middle cerebral artery (human)	Acute	1	N.R.	Guide wire	No complications reported	Recording	2 – 5× higher recording amplitudes than scalp EEG
Boniface and Antoun (1997)	Origin of middle cerebral artery (human)	Acute	1	Length: 20 mm	Guide wire	No complications reported	Recording	Impedance: 33 Ω Detected epileptiform seizures, higher recording amplitudes than scalp EEG depending on electrode location
Stoeter et al. (1995)	Middle meningeal artery, basilar artery, callosomarginal artery, middle cerebral artery (human)	Acute	1	Length: 3 – 60 mm	Guide wire	No complications reported	Recording	Impedance: 50 Ω Higher recording amplitudes than scalp EEG depending on electrode location
Mikuni et al. (1997)	Cavernous and superior petrosal sinus (human)	Acute (3 – 40 h)	1	Length: 30 mm Diameter: 0.28 mm	Guide wire	Headaches during procedure; no other complications	Recording	Impedance: 20 – 40 kΩ Detected epileptiform spikes not detected through scalp EEG
Llinás et al. (2005)	Sciatic artery and spinal cord arteries (N.R.)	Acute	4	Diameter: 20 μm	Nanowire array	No complications reported	Recording	Different electrodes recorded different amplitudes Higher recording amplitudes than electrodes on the spinal cord surface
			2 – 4	Diameter: 0.6 μm				
Bower et al. (2013)	Superior sagittal sinus (swine)	Acute	4 macroelectrodes, 16 microelectrodes	Macroelectrode length: 1 mm Microelectrode diameter: 40 μm	Straight catheter	No complications reported	Recording	Comparable performance to subdural ECoG Detected high-frequency neural activity
Gaba et al. (2016)	Vein of Galen, straight sinus, petrosal sinus, occipital vein (baboon)	Acute	6	N.R.	Balloon catheter	Thrombus formation, small venous dissection	Recording and stimulation	Detected epileptiform seizures Neural tissue activation (0.3 – 20 mA threshold) Catheter breakage
			8	Length: 1 mm	Basket catheter			
Nicolai et al. (2023)	Internal jugular vein (swine)	Acute (8 – 10 h)	8	Surface area: 1.2 mm^2^	Basket catheter	No complications reported	Stimulation	Nerve activation (860 μA threshold)
Chen et al. (2022)	Femoral artery (swine)	Acute	1	N.R.	Lead wire	No complications reported	Stimulation	Nerve activation (3 V) Wireless power transfer of 4 mW
Iwata et al. (2024)	Superior sagittal sinus, cortical veins, deep veins (swine)	Acute (5 – 6 h)	2	Length: 5 mm	Guide wire	No complications reported	Recording and stimulation	Resistance: 1 kΩ PSD exceeds noise floor up to gamma range Neural tissue activation (20 mA)
Masuda et al. (2025)	Bilateral transverse sinuses, bilateral cavernous sinuses, superior sagittal sinus (human)	Acute (50 min)	6	Length: 3 mm Diameter: 0.25 mm	Multiple micro wires	No complications reported	Recording	3 – 4x higher recording amplitudes than scalp EEG More sensitive than scalp EEG up to 60 Hz

To enable spatial localisation of recorded signals, subsequent work developed more complex device architectures focusing on miniaturisation and increasing electrode numbers. An electrode array comprising four 20 μm diameter wires was developed for peripheral nervous system (PNS) recordings of the sciatic nerve via implantation in the sciatic artery (Llinás et al., 2005). Different electrode contacts were found to record signals differing in amplitude, which suggests feasibility for spatial localisation. This work also presented the first instance of spinal cord recordings, employing 0.6 μm diameter nanowire electrodes. Endovascular electrodes recorded higher amplitude signals than electrodes located on the surface of the spinal cord. As in the brain, endovascular electrodes in the spinal cord may be closer to the target tissue than arrays placed on the surface. This report also proposed the metal wires could be replaced with softer, more malleable conducting polymer (CP) nanowires made of polypyrrole (PPy). This approach was expected to enhance steerability and facilitate routing to smaller diameter vasculature. In 2013, Bower et al. (2013) further increased electrode numbers by developing a catheter with 4 macroelectrodes and 16 microelectrodes for implantation in the SSS of pigs. This device successfully detected penicillin-induced cortical seizures with similar signal amplitude as subdural electrode arrays. Endovascular electrodes recorded signals across a broad range of frequencies and were able to detect activity seemingly arising from small neuron populations, suggesting that the device could be successful in locating epileptic foci. This study exemplifies the growing shift towards venous implantation, where reduced smooth muscle content, blood pressure and flow velocity may mitigate risks of vessel piercing and uncontrolled haemorrhage, as well as noise artifacts (Guyton, 2016; Wolfe and Kress, 2016).

Beyond the more established recording abilities, ENIs have also expanded into stimulation applications. In 2016, endovascular catheters were used to access various locations in the venous system of baboons, contact the vessel wall, detect abnormal signals and activate cortical tissue by stimulation (Gaba et al., 2016). This was a proof-of-concept study to show that the technology could be used to treat refractory epilepsy. The study compared two commercial catheters and two novel catheter prototypes. The novel catheters consisted of a Pebax balloon catheter mounted with six electrodes, and a nitinol basket catheter mounted with eight platinum-iridium electrodes (Figure 5A). These were implanted in the petrosal sinus, straight sinus, vein of Galen and occipital vein, and used to target multiple cortical structures including the occipital lobe, temporal lobe and parietal lobe. Although only acute studies were conducted, epileptic seizures were successfully detected, and activation of tissue was achieved in most cases with current thresholds between 0.3 and 20 mA. The novel balloon catheter achieved the lowest thresholds, presumably due to improved contact with the vessel wall. This showed ENIs are promising platforms for brain stimulation, and more recently they have also been trialled for PNS applications (Nicolai et al., 2023). Vagus nerve stimulation has been proposed as a treatment for various neurological disorders including epilepsy, depression, and anxiety. However, stimulation using standard nerve cuff electrodes results in multiple off-target effects. An endovascular approach could minimise these, since it enables implantation more caudally, after further branching. This location is incompatible with nerve cuff implantation since it would require a significantly more invasive and higher risk procedure. To investigate this, a commercial basket catheter was implanted in the internal jugular vein for vagus nerve stimulation. Compound action potentials were successfully induced upon stimulation at 860 μA, whereas current thresholds for nerve cuffs remained significantly lower at 300 μA. Nerve activation was also highly dependent on electrode positioning, highlighting the importance of electrode visualisation during implantation. The authors noted that a custom electrode array would have to be developed for long-term implementation of this technique, as the commercial catheter employed is not indicated for chronic use. Endovascular stimulation has also been employed for acute femoral nerve stimulation in swine models (Chen et al., 2022). Here, a 3 V monophasic stimulus pulse at 10 Hz induced compound nerve action potentials that could be recorded by electrodes downstream. In this case, the device consisted of a wireless magnetoelectric implant that bypassed the need for connecting wires, delivering up to 4 mW power at a transmission depth of 4 cm. However, due to the large size of the proof-of-concept device, it could only be implanted adjacent to the blood vessel and was connected to a wire inserted in the vessel which delivered the stimulation (Figure 5B). Upon miniaturisation for placement within the vessel, the device would require a tethering mechanism to avoid movement due to blood flow.

**Figure 5:**
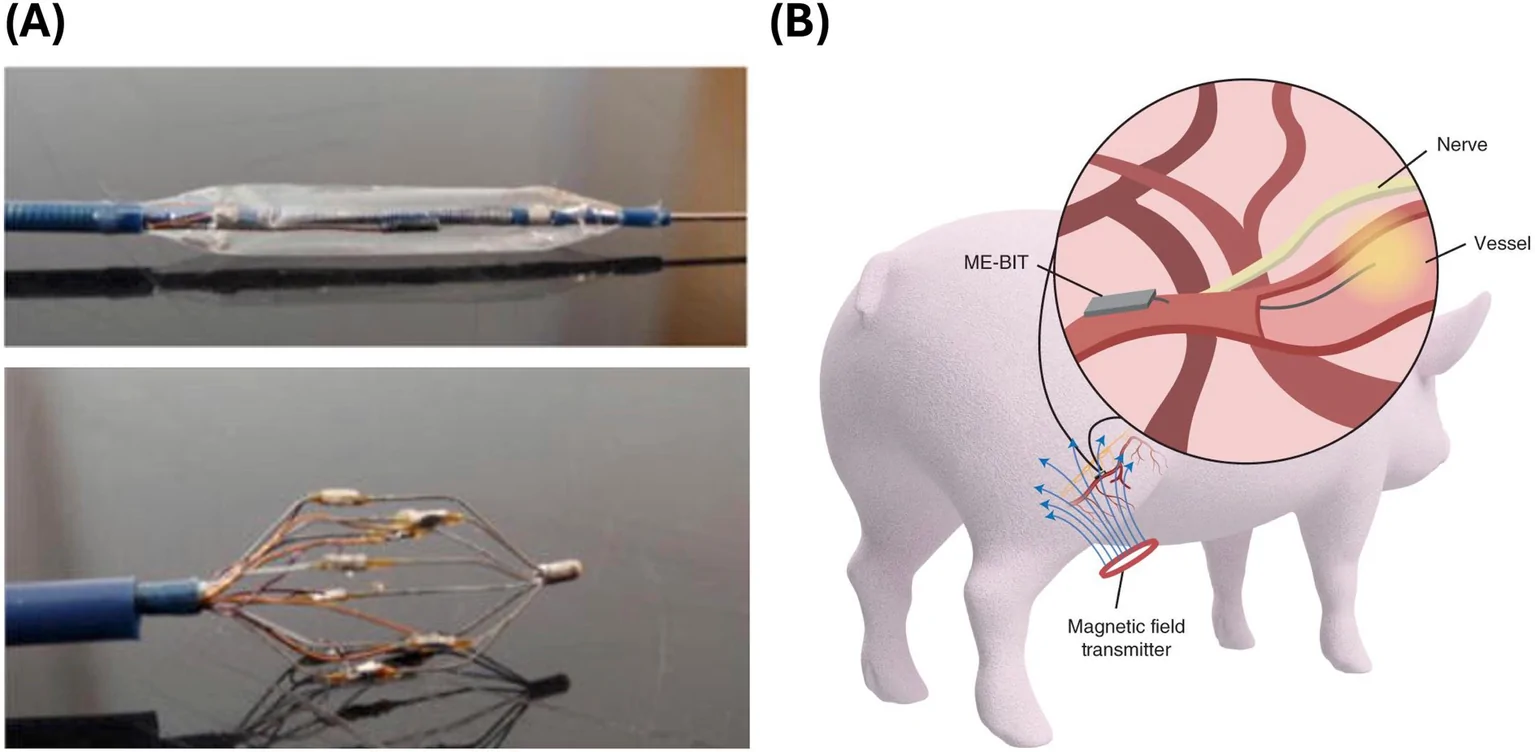
Novel catheter prototypes for venous pacing in the CNS. **(A)** Top: Over-the-wire balloon catheter. Pebax balloon mounted with six electrodes in a helical pattern. Bottom: Basket catheter made of nitinol with Platinum/iridium electrodes. Adapted from Gaba et al. (2016). Used under CC BY 4.0. **(B)** Schematic of magnetoelectric implant for endovascular peripheral nerve stimulation. The proof-of-concept device is implanted outside the vessel due to its larger dimensions, and is connected to a lead that enters the femoral artery to deliver stimulation. Adapted from Chen et al. (2022). Used under CC BY 4.0.

After proving the feasibility of endovascular recording and stimulation of neural tissue, research focused on improving the signal quality achievable. Electrodes located closer to the target tissue generally exhibit higher spatial resolution. Thus, the spatial resolution achievable by an ENI is likely to be correlated to the vessel wall thickness and composition at the location of implantation. As wall thickness is proportional to vessel diameter, Iwata et al. investigated whether clearer signal recordings from a greater range of brain regions could be obtained by measuring cortical activity from small and deep veins in comparison to larger veins (Iwata et al., 2024). For this, micro guide wires used as electrodes were placed in cortical veins as small as 0.5 mm in diameter and in the SSS (approximately 2 mm in diameter) in pigs. Cerebral venous sinuses such as the SSS differ in structure from cerebral veins: sinuses are located between two layers of the dura mater, in a channel lined with endothelial cells. On average, the dura is 0.36 mm thick, whilst cortical vein walls are almost 10 times thinner (Fam et al., 2018; Bateman and Bateman, 2025). Predictably, signals obtained from the cortical veins showed a greater SNR than recordings from the SSS, as well as a greater ability to detect localised activity. The study also showed the ability to elicit motor evoked potentials in lip and shoulder muscles through electrical stimulation at the cortical veins. Additionally, implantation of guide wires in deep veins allowed recording of visual evoked potentials from the occipital lobe, which were undetectable when recording from more distant cortical veins. Depending on the diameter and wall thickness of the vessel, high resolution neural recording and interfacing can be achieved with deep brain structures that are normally inaccessible to dense electrode arrays.

ENIs have proved to be successful in recording neural activity from a wide range of cerebral regions. More recently, the possibility of recording signals from multiple brain regions simultaneously has also been explored (Masuda et al., 2025). Accurate localisation of epileptic foci requires recording neural activity from large brain areas with high spatial resolution. For this, 6 thin, flexible EEG electrodes were implanted in 6 distinct cerebral sinus locations. Although no seizures occurred during the duration of this acute experiment, intravascular EEG recordings showed amplitudes 3-4 times higher than scalp EEG, similar to what has been reported before. Notably, this work introduces the possibility of deploying multiple ENIs in multiple brain locations for minimally invasive recording or stimulation of the entire brain with higher spatial resolution.

Taken together, these studies demonstrate the feasibility of employing ENIs both as recording and as stimulating tools that outperform non-invasive EEG, with evidence to indicate further improvements when targeting vessels with thinner walls. However, these devices lack the stability required to record signals chronically. Adaptations of existing catheters and micro guide wires are used, which are not designed for long-term implantation and lack any anchoring or stable contact to the vessel wall. Thus, the recordings are prone to pulse artifacts and noise due to blood flow, or to being displaced from the target location. To solve this issue, Synchron introduced the Stentrode™ in 2016, establishing the form factor of SEAs (Oxley et al., 2016). By integrating the electrodes on a stent, SEAs can directly anchor to the vessel wall, resulting in a more stable interface.

### 3.2 Stent-electrode arrays

#### 3.2.1 The Stentrode™

The Stentrode™ represented a landmark development in the field of ENIs, receiving FDA breakthrough device designation in 2020 (Oxley et al., 2016; Han, 2021). Briefly, the device consists of a 3 mm self-expanding nitinol vascular stent (Solitaire SAB, Covidien), mounted with platinum electrodes and connected via a transvascular lead to an implantable receiver transmitter unit (IRTU) that wirelessly communicates to an external device (Figure 6). The Stentrode™ was designed for deployment in the SSS to record and decode neural activity from the adjacent anterior bank of the motor cortex (Oxley et al., 2016). Implantation of the array was achieved by compressing it into a 4F catheter (outside diameter: 1.33 mm) and guiding it using angiographic delivery with the aid of pre-operative MRI venograms. In the implantation procedure, the catheter carrier was inserted into the jugular vein and guided through the venous system to reach the SSS where the Stentrode™ was deployed and the catheter was subsequently removed. The transvascular lead was then sutured at the puncture site in the jugular vein and subcutaneously channelled to the sternocleidomastoid, where a connector coupled it to the signal acquisition system.

**Figure 6:**
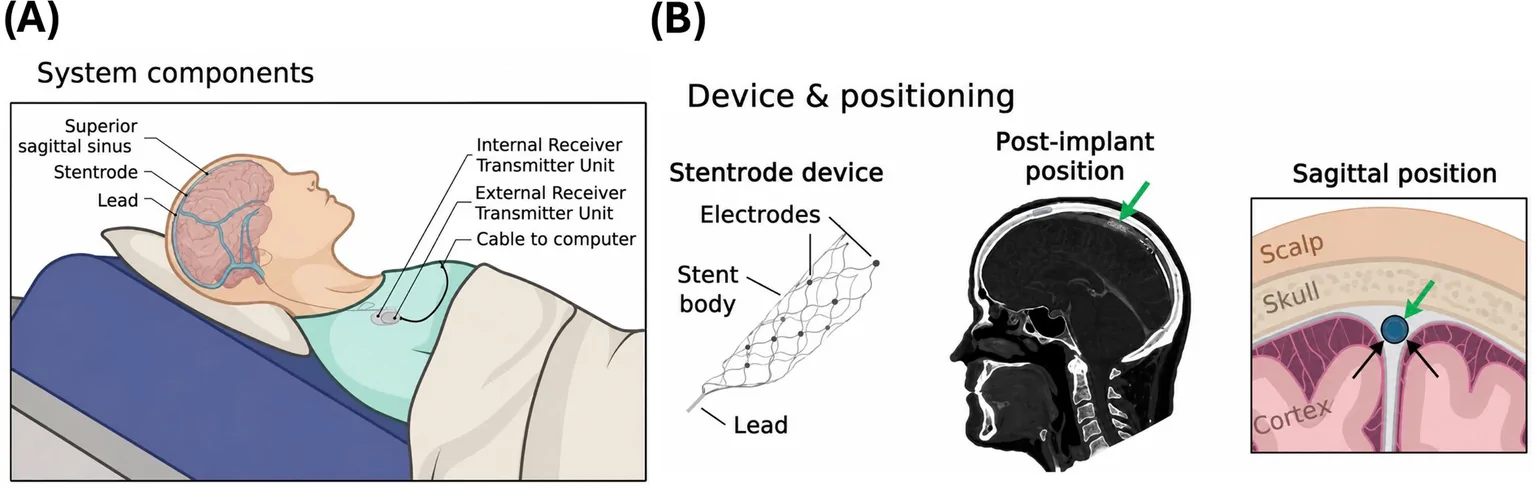
**(A)** Schematic of the Stentrode being employed as a brain-computer interface. The device is implanted in the Superior Sagittal Sinus, adjacent to the Motor Cortex. A lead connected to the device travels through the vasculature to the Internal Jugular Vein, where it exits vasculature and connects to an Internal Receiver Transmitter Unit (IRTU) implanted subcutaneously in the chest. An External Receiver Transmitter Unit connects the IRTU to a computer. **(B)** Device and positioning upon implantation. From left to right, an image of the Stentrode, composed of the stent body, electrodes, and connecting lead; CT image showing the positioning of the Stentrode in the SSS (green arrow) upon implantation; schematic of the positioning of the Stentrode in a sagittal plane, located in the SSS (green arrow) between the two hemispheres of the brain, where it records signals (black arrows) from the Motor Cortex. Adapted from Chetty et al. (2025). Used under CC BY 4.0.

The device enabled chronic recording of motor cortical activity for up to 190 days. Some electrodes failed due to wire breakages triggered by the neck movement of the animal owing to the use of delivery wires that are not indicated for chronic use. Additional failures also occurred due to short circuiting of the electrode contacts to the metallic stent. Signal quality improved over the first 28 days following device implantation, after which recording amplitudes remained constant. Studies found electrode impedance decreased at low frequencies in the first 8 days, whilst phase angles increased, suggesting alterations of the electrode-tissue interface (Opie et al., 2016). Histological and micro-CT assessment confirmed that most stent struts were covered in neointimal growth by 2 weeks post-implantation, showing that endothelialisation occurred on a similar timeline to cardiovascular stents (Opie et al., 2017). Incorporation of the ENI into the vessel wall is desirable as it prevents device migration, decreases the distance between the electrodes and neural tissue, stabilises recordings and reduces the risk of thrombosis. No vessel occlusion was observed, although some lumen reduction was seen around the device components in areas with greater variations to haemodynamics (John et al., 2022). Computational results showed that oversized stents could have a greater impact on haemodynamics and tissue remodelling, and that neointimal proliferation in healthy vessels may be different to diseased vessels (Qi et al., 2024). Overall, studies found no evidence of neointimal hyperplasia or neurological consequences associated with disrupted vascular flow, showing the Stentrode™ is compatible with safe, long-term implantation.

Acute intracranial recording capabilities from the SSS were assessed in ovine models by placing the Stentrode™ adjacent to the motor cortex and triggering somatosensory evoked potentials through direct electrical stimulation of the median nerve (Oxley et al., 2016). Different electrodes obtained signals from seemingly distinct neuron populations, showing potential for signal localisation. Power spectra and bandwidth were found to be comparable to commercial epidural and subdural ECoG arrays. The subdural ECoG array performed marginally better than the Stentrode™, which was to be expected due to increased electrode-to-target distance arising from the dural layer separating the lumen of the SSS from the cerebral cortex. However, subsequent studies suggest that these results may differ for chronically implanted devices (John et al., 2018). A later timepoint would allow for endothelialisation of the Stentrode™, but coincides with fibrous encapsulation of the ECoG arrays which would increase electrode impedance and degrade signal quality. Comparison of the Stentrode™ to ECoG arrays after chronic implantation for 2 weeks found that the subdural array outperformed the Stentrode™ only in spatial resolution at low frequencies (< 25 Hz). Achieving this spatial resolution endovascularly may be possible but would require smaller electrodes and reduced electrode-to-target distance.

The success of the Stentrode™ as a recording tool in animal trials led to its first in-human study, a Stentrode™ with thought controlled digital switch (SWITCH), with results published in 2023 (Mitchell et al., 2023). Here, the Stentrode™ was implanted in the SSS in four patients with severe upper-limb paralysis to record neural activity from the motor cortex. A transvascular lead connected the device to the IRTU, which was placed subcutaneously under the clavicle. In the 12 months following device implantation, no serious adverse events were reported. There was no thrombosis, vessel occlusion or significant movement of the device after implantation. The recording bandwidth of the device remained stable during the 12 months, and between 5 and 7 attempted limb movements could be decoded from the signal recordings for all patients. Together with an eye tracking system, this allowed patients to control a computer to email, text, or perform financial tasks, regaining independence and ability to communicate. Currently, the Stentrode™ is undergoing another clinical trial with similar endpoints in the US, the COMMAND trial (Chetty et al., 2025).

Following its success as a chronic brain computer interface (BCI) for recording applications, the Stentrode™ was subsequently investigated for neural stimulation. This was first trialled in 2018 for stimulation of the motor cortex in sheep (Opie et al., 2018). The same 3 mm diameter device was deployed in the SSS at the intersection with the cruciate sulcal veins where the sinus has a diameter of 1.6 mm and marks the commencement of the motor cortex in sheep (John et al., 2017; Oxley et al., 2017). In this case, the percutaneous connector was placed on the back of the animal to minimise the risk of mechanical failure. Stimulation started 28 days after implantation, to allow complete endothelialisation of the device and ensure signal stability. The stimulation current was adjusted in each case to induce muscle activity, without exceeding 10 mA or the charge injection capacity (CIC) of the electrodes. This study found that the electrode location was directly correlated to the elicited response, with electrodes at different distances from the cruciate sulcal veins causing distinct responses such as lip movements or facial responses. The efficacy of the Stentrode™ was compared to that of subdural electrodes (4 mm stainless steel ball or subdural platinum disk electrode grids) and penetrating electrodes (1 mm diameter, 3 mm length stainless steel needle electrodes). These control devices were also able to elicit a response in most cases, but no strong correlation was found between electrode location and activated muscle. Regardless of electrode position, similar current thresholds between 4.5 – 6 mA were sufficient to evoke all the distinct motor responses. Notably, electrodes facing the skull exhibited higher current thresholds than those oriented towards the brain. Moreover, almost half of the electrodes failed to elicit a response. It was later reported that the high currents required in some cases to achieve motor cortex activation would have required surpassing the safe CIC of the electrodes (Harris et al., 2025). The limited CIC of platinum electrodes is a significant drawback to the present device iteration as extended charge injection above this limit can generate toxic electrochemical species and damage the electrode itself (Cogan, 2008). To address the recurrent difficulties in achieving effective stimulation, a recent study attempted to increase the CIC of the electrodes on the Stentrode™ by increasing their electroactive surface area (Harris et al., 2025). By modifying the platinum electrodes with sputter coated platinum black, the roughness of the electrodes was increased, thus resulting in decreased impedance and a three-fold increase in safe CIC (from 21.9 μC cm^−2^ to 64.9 μC cm^−2^). Computational modelling results showed that platinum black doubled the maximum distance at which neurons could be activated from 0.58 to 1.14 mm, although it remains to be proven whether these improvements are sufficient to allow safe stimulation of neural tissue *in vivo*.

In addition to the SSS, the Stentrode™ was also trialled for endovascular stimulation of the visual cortex when implanted in the transverse sinus (Gerboni et al., 2018). Stimulation resulted in visually evoked potentials that were detectable from a subdural array. Recordings from the subdural array suggested that electrodes on the Stentrode™ elicited a localised response, with those located more anteriorly on the device being more likely to achieve detectable activation. The threshold current for neural activation was found to be 2 mA, corresponding to an injected charge of 362.2 μC cm^-2^, which is notably greater than the CIC values reported by Harris et al. (2025). More recently, the Stentrode™ has also been trialled for stimulation in the PNS (Liu J. et al., 2024). The device was implanted in the femoral artery in sheep for acute femoral nerve stimulation, and its performance was compared to that of a commercially available pacing catheter. Results showed that stimulation of the quadriceps could be achieved without surpassing the safe CIC of the platinum electrodes. The current thresholds for stimulation with the SEA were twice as high as those required by the pacing catheter. However, the small sample size and inability to orient the electrodes on the stent towards the target nerve could explain these discrepancies. It was also suggested that larger electrodes with superior charge injection abilities might be required to achieve a functional response, as the CIC is proportional to electrode surface area.

The Stentrode™ has been shown to be effective as a BCI when implanted in the SSS, with the potential to revolutionise the quality of life of patients suffering from paralysis or locked-in syndrome. Long-term assessment of the device shows capabilities for chronic recording applications, and initial results prove feasibility of stimulation.

#### 3.2.2 Recent developments in SEAs

The Stentrode™ introduced the concept of stent-based ENIs to prioritise stable wall contact and long-term performance. Subsequent work has carried this forward with the use of stent-like meshed structures for vessel apposition, whilst focusing on targeting alternative implantation locations and enhancing device charge injection abilities through a range of strategies (Table 4).

**Table 4:** Collection of the key parameters and characteristics of studies on SEAs (N.R., not reported; N.A., not applicable).

Citation	Implantation vessel (subject / model)	Implantation duration	Electrode number	Electrode size	Form factor	Safety outcomes	Recording or stimulation	Performance metrics
Oxley et al. (2016)	Superior sagittal sinus (sheep)	Chronic (up to 190 days)	6–8	Diameter: 750 μm	Stent-electrode array (Stentrode)	Subdural haemorraghe (*n* = 1 of 22) Occlusion of 37% of cortical veins entering at the point of timplantation; no clinical sequelae	Recording	Impedance: 2.66 ± 0.5 kΩ at 10 kHz Spatial resolution ≥ 2.4 mm Recording bandwidth: 189 ± 6 Hz Electrode failure due to shorting and wire breakage
Mitchell et al. (2023)	Superior sagittal sinus (human)	Chronic (1 year)	16	Surface area: 0.3 mm^2^	Stent-electrode array (Stentrode)	Mild adverse effects: bruising at incision sites, hematoma at insertion site, anemia, postoperative fatigue	Recording	Impedance: 37 ± 11 kΩ Recording bandwidth: 233 ± 16 Hz
Opie et al. (2018)	Superior sagittal sinus (sheep)	Chronic (up to 6 months)	6–12	Diameter: 500 – 750 μm	Stent-electrode array (Stentrode)	No complications reported	Stimulation	Impedance: 1 – 20 kΩ Neural tissue activation (4.5 ± 0.87, 5.63 ± 0.41, 5.89 ± 1.76 and 5.7 ± 0.34 mA threshold, 200 – 300 μs pulse to elicit different responses)
Gerboni et al. (2018)	Transverse sinus (sheep)	Acute	8	Diameter: 750 μm	Stent-electrode array (Stentrode)	No complications reported	Stimulation	Impedance: 1 – 30 kΩ at 1 kHz Neural tissue activation (2 mA estimated threshold, 800 μs pulse)
Liu J. et al. (2024)	Femoral artery (sheep)	Acute	4	Diameter: 750 μm	Stent-electrode array (Stentrode)	No complications reported	Stimulation	Nerve activation (1 ± 0.11 mA threshold, 500 μs pulse)
Wen et al. (2025)	Superior sagittal sinus (rat)	Acute	4	Surface area: 0.157 mm^2^	Stent-electrode array	Thrombus formation Tissue damage	Recording	Impedance: 2 kΩ (in vitro), 12.8 kΩ (in vivo) SNR: 3.1 dB Effective bandwidth: 18 – 32 Hz
Zhang et al. (2023)	Middle or anterior cerebral artery (rat)	Chronic (28 days)	16	Diameter: 80 μm	Stent-electrode array	No complications reported	Recording	Single neuron spike detection
Fanelli et al. (2022), Diao et al. (2025)	No *in vivo* data	N.A.	4	Diameter: 600 μm	Stent-electrode array	N.A.	Recording and stimulation	Impedance: 5.5 ± 3 kΩ Charge injection capacity: 5.2 ± 4.8 mA
Diao et al. (2025)	No *in vivo* data	N.A.	16	N.R.	Stent-electrode array	N.A.	Recording	Impedance: Approximately 1 kΩ

Implantation in smaller vasculature calls for miniaturised devices. An SEA fabricated by attaching laser ablated microwires to a Solitaire AB commercial stent was developed for implantation in the SSS of a rat (1–2.5 mm diameter) (Wen et al., 2025). The device featured microelectrodes as small as 25 μm in diameter, and the study reported obtaining a superior SNR and spatial resolution to the Stentrode™. However, it should be noted that this was assessed by recording pulse signals delivered from wires placed within the blood vessel rather than neural activity. They experienced clotting issues and tissue damage, presumably due to the stent being both significantly larger in diameter than the blood vessel and more rigid. This underscores the importance of tailoring device design to the implantation location.

One strategy to mitigate the risk of damage to vasculature is the use of softer materials. Polymers are a promising candidate for this and are already becoming increasingly popular in novel medical implants, stents and NIs. Being several orders of magnitude softer than metals, polymeric materials reduce mechanical mismatch with tissue, decreasing the risk of tissue damage and fibrous encapsulation (Cuttaz et al., 2019). In 2018, a polymeric SU-8 based probe was developed for endovascular neural recording from micrometre-scale vasculature in rats (Zhang et al., 2023). Photolithography techniques were used to fabricate a mesh-like structure with a strut thickness of 0.8 μm, and 16 platinum electrodes (80 μm diameter) were mounted on the mesh (Figure 7A). The device was implanted in blood vessels under 100 μm in diameter using a microcatheter and saline flow. Penicillin-induced seizures were successfully detected and located. The thin vessel walls also allowed detection of single unit spikes, serving as a first-time demonstration that ENIs can record single-cell activity and exhibit a spatial resolution comparable to that of microelectrode arrays implanted in direct contact with the brain (Liu K. et al., 2024). Histological examination after 28 days showed no neointimal formation or significant immune response, showing that soft polymeric materials could be less likely to trigger a reaction than metals. Although no adverse effects were reported in this study, SU-8 generally exhibits low haemocompatibility and alternative materials might be more appropriate for translation of this technology (Chen and Lee, 2021).

**Figure 7:**
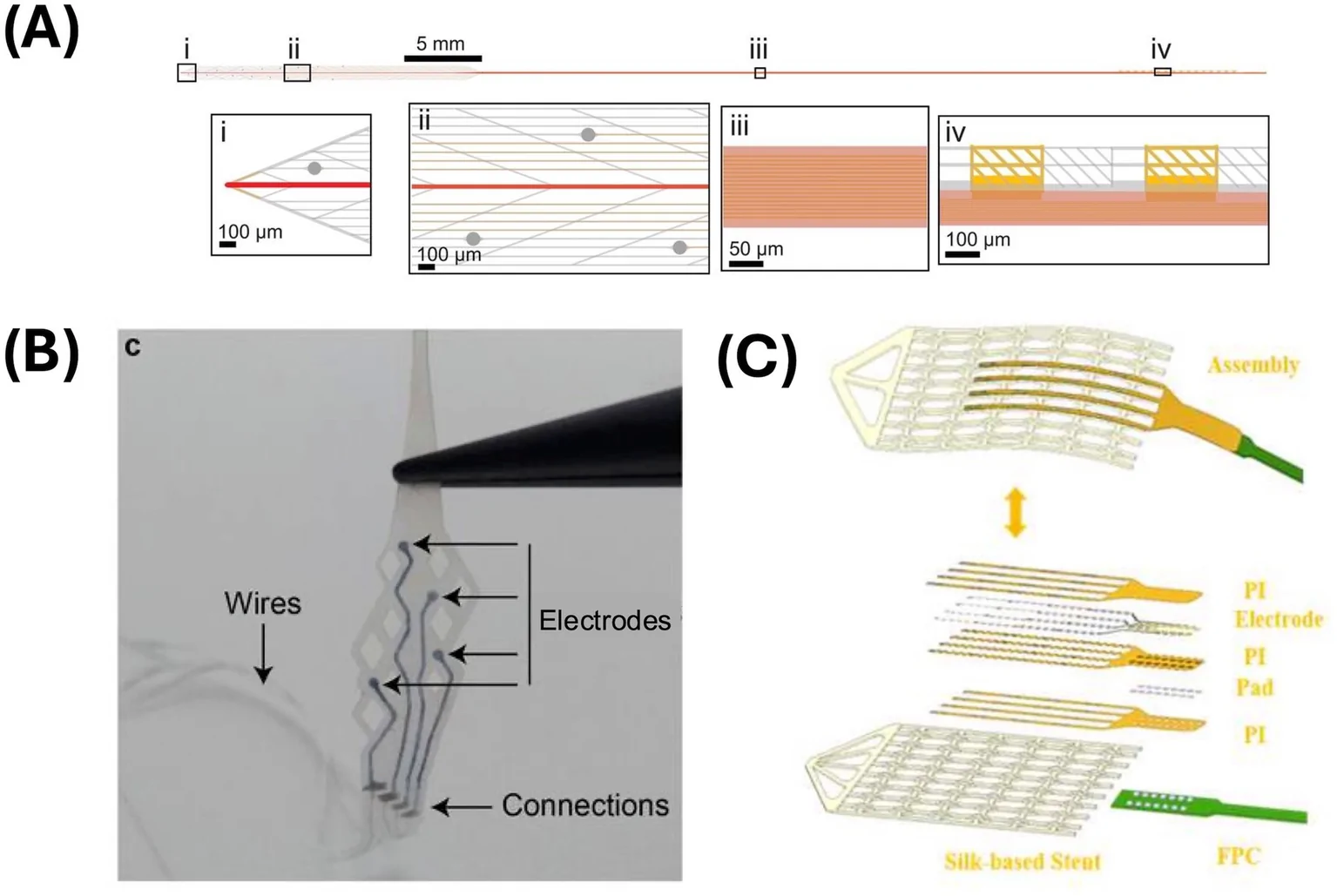
**(A)** Ultraflexible microendovascular probe. Insets zoom into the probe, showing the SU-8 guidewire in red, SU-8 mesh region in grey, platinum electrodes as grey circles, and gold connecting pads in yellow. Modified from Zhang et al. (2023). Copyright © 2023 The Authors. **(B)** Fully polymeric, transient neurovascular interface. Electrodes made of PEDOT: PSS integrated in a PCL scaffold, fabricated via moulding and laser cutting techniques. Adapted from Fanelli et al. (2022). Used under CC BY 4.0. **(C)** Schematic of silk fibroin-based stent-electrode array. Layered structure shows polyimide-insulated electrode array attached to silk-based stent and connector cable. Adapted with permission from Diao et al. (2025). Copyright © 2025 IEEE.

Polymers could also be employed to address the issue of suboptimal charge injection. This was demonstrated with the development of a fully polymeric transient SEA in 2021 (Fanelli et al., 2022). Here, the electrical components were made of the CP poly (3,4-ethylenedioxythiophene):poly(styrene sulfonate) (PEDOT: PSS), whilst the main stent scaffold was made of polycaprolactone (PCL) (Figure 7B). PEDOT: PSS was employed due to its high CIC in aqueous environments and softer mechanical properties. Fabricated using moulding and laser cutting techniques, the high CIC values reported indicate suitability for stimulation applications. Although no *in vivo* or *ex vivo* experiments were conducted, implantation in mock vessels of 2 mm in diameter revealed good apposition to the vessel wall. However, the fabrication techniques employed do not allow for further miniaturisation of the device, and its intended degradability *in vivo* restricts its use to short-term applications.

Another example incorporating degradability employed a silk fibroin stent as the scaffold to attach metallic electrodes and tracks insulated with polyimide (Diao et al., 2025; Figure 7C). In this case, the stent scaffold would degrade over time leaving the insulated electrode array behind. Further efforts investigated the possibility of incorporating drugs into the stent scaffold for gradual release as the scaffold degrades. This imitates the approach of drug-eluting stents, which consist of a stent body coated in blends of degradable polymers and antiproliferative drugs that are slowly released to reduce the risk of neointimal hyperplasia. The study characterised silk fibroin as a bulk material, but not in stent form. Since it is not a material commonly used in stents, ensuring sufficient mechanical strength and haemocompatibility will be important for translation. The electrode contacts were also surface treated by electrodeposition of PEDOT/PSS to decrease impedance and increase CIC. Overall, recent advancements exhibit a trend towards greater device complexity, with a focus on exploring novel materials to enable miniaturisation, enhance stimulation abilities, and reduce mechanical stiffness.

## 4 Discussion

ENI development has been heavily interlinked with advances in interventional cardiology which has established core design and use principles (Figure 8). To ensure chronic vascular safety, ENIs can adopt designs, material selection and testing protocols developed for cardiovascular stents. Secondly, smart stents introduced the possibility of wireless power transfer to stents, which has more recently been explored for ENIs specifically. Collaboration in this aspect would accelerate the development of this technology. Additionally, fabrication methods employed for smart stents would enable the development of complex ENIs, as well as the integration of sensors for more advanced monitoring of implant health, intracranial pressure or haemodynamics. Conversely, ENIs provide an additional use case for the technologies that have been developed for smart stents, which largely remain limited to *in vitro* or ex vivo studies. The use of cardiovascular catheters for stimulation has demonstrated the feasibility of endovascular peripheral nerve stimulation, introducing a range of applications for ENIs in the autonomic nervous system. The use of different form factors such as basket, lasso or spiral catheters could also be translated to ENIs. In turn, the electrochemical characterisation and optimisation of stimulation parameters performed in ENI research can inform the practice in cardiovascular applications for treatment and management of chronic cardiovascular conditions.

**Figure 8:**
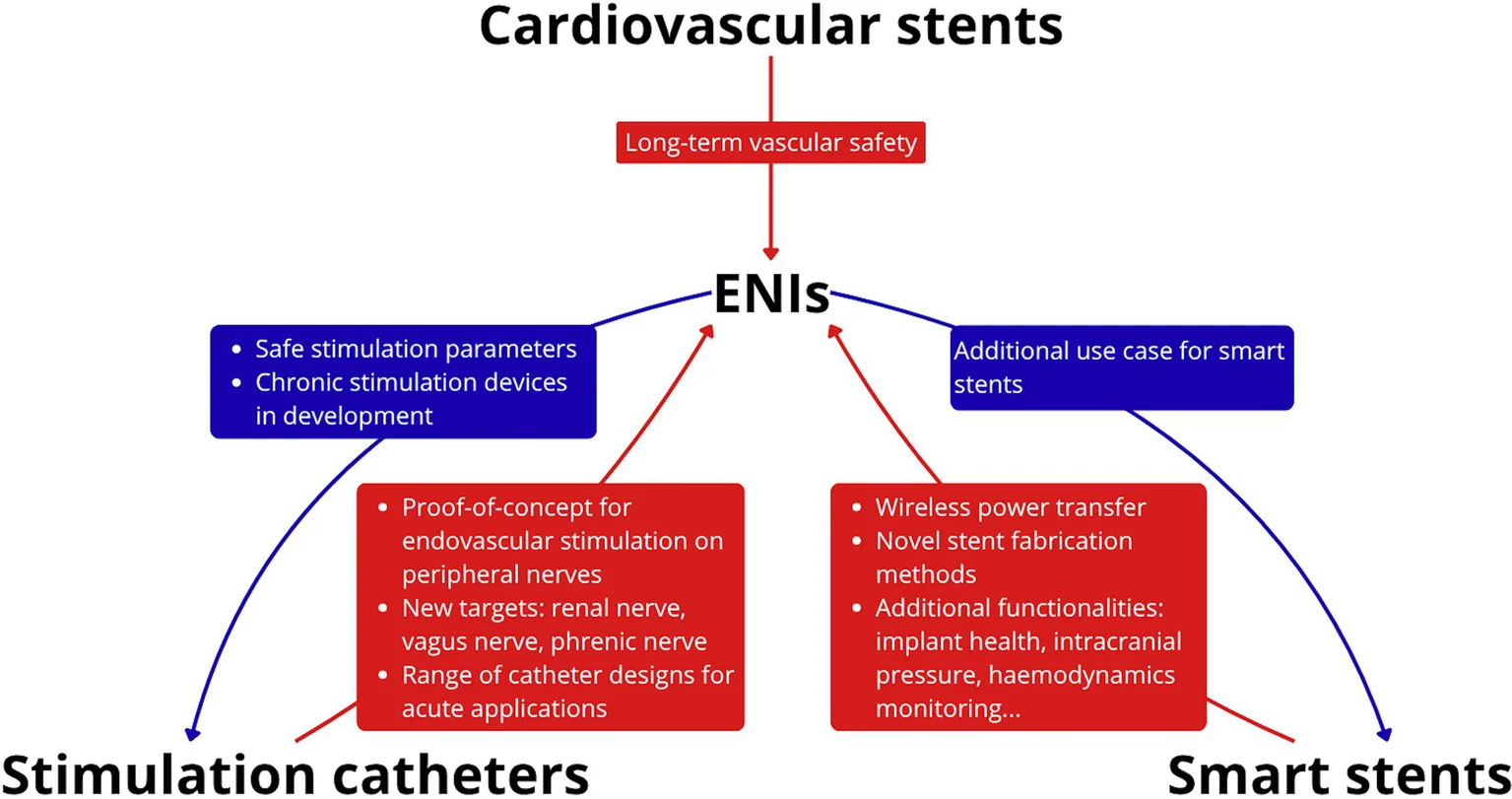
Overview linking ENIs to cardiovascular technologies. Red pathways indicate cardiovascular developments that may aid ENI design and safety; blue pathways indicate areas in which ENI developments could enhance the progress of cardiovascular technologies. Figure produced using Canva.

### 4.1 Key engineering challenges

The existing research on endovascular bioelectronics spans a wide range of capabilities, applications and form factors. A substantial amount of proof-of-concept work has been established, yet further development and consolidation is required before these technologies can be realised into treatments for the full range of conditions they may benefit. To date, it has been proven that endovascular devices can record neural activity with a sufficient SNR to decode information (Mitchell et al., 2023). Recordings from smaller blood vessels have shown sufficient spatial resolution to capture single-cell activity (Zhang et al., 2023). Endovascular stimulation can activate neural tissue, and although it requires higher currents than within tissue stimulation, this trade-off is justified by the reduced risk of neural tissue damage and minimally invasive implantation (Opie et al., 2018; Iwata et al., 2024; Liu J. et al., 2024). The integration of electrode arrays on a stent marked an important milestone, with the development of SEAs providing a chronically stable platform for endovascular neural interfacing (Oxley et al., 2016). Upcoming research efforts are expanding the applications of ENI technology through device miniaturisation, enhancing stimulation abilities, and incorporation of wireless communication systems, whilst ensuring long-term vascular safety.

#### 4.1.1 Device miniaturisation

It has been shown that greater spatiotemporal resolution can be achieved when ENIs are deployed in smaller blood vessels with thinner walls (Iwata et al., 2024). This requires miniaturisation of devices and electrodes, which carries additional considerations due to their vascular deployment. Firstly, small, thin-walled vessels are more prone to piercing or damage leading to neointimal hyperplasia. Thus, mechanical congruence with the vessel wall becomes key when miniaturising the device. Secondly, device thickness must decrease proportionally with diameter to maintain vessel patency. To fulfil these requirements, alternative fabrication methods and materials must be considered.

The first generation Stentrode™ consisted of platinum electrodes welded to wires and mounted on a commercial stent using an adhesive (Oxley et al., 2016). To feature smaller microelectrodes, higher electrode densities and more seamless integration of the electronics and the stent, the use of more automated, precise thin film microfabrication techniques common in conventional NI fabrication will be required. These fabrication methods include laser fabrication, 3D printing and photolithographic processes, and have already been employed in active stents. Oyunbaatar et al. (2023) showed that photolithography techniques can be used to fabricate a stent integrated with a pressure sensor; a similar methodology would allow incorporation of a high density of microelectrodes onto a custom-design stent. Zhang et al. (2023) demonstrated this by using photolithography to fabricate a probe featuring the smallest electrode size in an ENI to date. Integration of high electrode densities and sensors with additional functionalities onto stent-like structures will enable the development of advanced ENIs that can be self-reporting or allow closed-loop neuromodulation.

Regarding material choice, ENIs are likely to benefit from employing softer materials that reduce mechanical mismatch with tissue, a common issue in neural interfacing (Cuttaz et al., 2019). This is especially important as the field transitions towards the SEA form factor, where stiff metals are employed not only as electrodes or wires, but as an anchoring mechanism exerting force on the vessel wall. Oversized stents and stiff struts lead to greater wall shear stress variations, which have been found to be correlated to vessel wall thickening in SEAs specifically (Qi et al., 2024, 2025). The long-term complication risks of stent implantation are well-known, and this has resulted in the development of polymeric bioresorbable stents in the cardiovascular field, which fully degrade in the body after fulfilling their purpose (McMahon et al., 2018). The most common material for bioresorbable stents is poly(L-lactic acid) due to its biocompatibility, processability and well-known degradation and mechanical properties. Other materials such as poly(lactide-co-glycolide) (PLGA), desaminotyrosine polycarbonate polymer (PTD-PC) or poly(D, L-lactide) (PDLLA) have also been employed, albeit far less commonly. A main drawback of polymeric stents is their decreased radial strength in comparison with their metallic counterparts, which presents an issue in the context of reopening occluded atherosclerotic vessels (McMahon et al., 2018). However, this could make them a prime candidate for SEAs, where the function of the stent scaffold is to establish stable contact with the vessel wall whilst the device becomes endothelialised, rather than reopening the vessel. A biodegradable stent scaffold would also introduce additional considerations. The electrodes and interconnects must be designed as a standalone array that remains functional in the absence of the stent. However, if successful, this approach offers a promising paradigm. Once the scaffold degrades, the endothelialised electrode array would remain within the vessel wall, drastically reducing device footprint. Non-degradable polymers could be employed to conserve the benefit of reduced mechanical mismatch in a more simplified manner. The tunability of mechanical and degradation properties enables tailoring to specific applications; in SEAs, this would enhance adaptability to different vessel diameters and shapes whilst minimising damage.

A transition towards softer materials, both degradable and non-degradable, can already be observed in recently developed ENIs, which have used SU-8, PCL or silk fibroin as scaffold materials (Fanelli et al., 2022; Zhang et al., 2023; Diao et al., 2025). Degradable polymeric coatings can also be employed to incorporate antiproliferative or antithrombogenic drugs, as seen in bioresorbable and drug-eluting stents, to further mitigate the risk of neointimal hyperplasia or thrombogenesis. The introduction of novel materials will likely require bespoke manufacturing processes or significant adaptation of existing methods. Characterisation of mechanical properties and device performance must be accompanied with verification of chronic biocompatibility and haemocompatibility. Moreover, materials should be magnetic resonance imaging (MRI) compatible and allow visualisation of the electrode contacts during the implantation procedure. This would enable their precise orientation towards target tissue during implantation, minimising the current threshold for stimulation as well as the possibility of side effects due to off-target tissue stimulation.

#### 4.1.2 Enhancing stimulation abilities

Research on endovascular stimulation remains far more limited than work in recording applications, and the few works that have investigated it reported a recurring limitation. Electrodes were unable to deliver enough charge to achieve a clinically significant effect without surpassing their safe CIC (Liu J. et al., 2024; Harris et al., 2025). This issue, common in NIs, is further aggravated by the presence of the vessel wall, which increases the distance between the electrodes and target tissue and therefore the current thresholds for stimulation. Device miniaturisation further worsens the problem. Reducing device footprint and electrode size is necessary to access smaller blood vessels and improve spatial resolution, but smaller electrode surface areas also decrease the amount of charge that can be safely delivered. Thus, a balance must be achieved between electrode size and CIC.

The mechanism of charge transfer of traditional metallic electrodes inherently limits their stimulation capabilities (Cogan, 2008). This has resulted in the development of a range of strategies to improve electrode CIC and enable delivery of sufficient charge to activate neural tissue using microelectrodes. Some of these involve surface treatments to increase the electroactive surface area available for charge injection through laser roughening or coating with platinum black coating (Green et al., 2014; Harris et al., 2025). Materials such as iridium oxide have also been employed in NIs due to their improved charge injection abilities (Sun et al., 2023). Another approach that has gained a lot of attention lately is the use of CPs, organic conductors with high CIC and chemical stability (Berggren and Malliaras, 2019). CPs are a popular choice in neural interfacing applications due to their compliant mechanics, which reduce mechanical mismatch between electrodes and soft tissues, and their favourable charge injection behaviour in aqueous environments. Additionally, CPs exhibit a larger electrochemical surface area than smooth metals, increasing the area available for ionic exchange. Some of the most common CPs include polyaniline (PAni), PPy and PEDOT. In particular, the processability and chemical stability of PEDOT: PSS have established it as the material of choice for many applications (Green et al., 2008). PEDOT: PSS has been employed as a coating for metallic electrodes, which show decreased impedance magnitude, increased charge storage capacity and increased safe CIC, as well as a more stable phase angle across a range of frequencies (Green et al., 2012; Cuttaz et al., 2019). Examples of this include the silk fibroin SEA with PEDOT: PSS coated metal electrodes recently developed (Diao et al., 2025). However, CP coatings are often reported to delaminate *in vivo* upon electrical stimulation (Green et al., 2015). PEDOT: PSS has also been used as a standalone material for electrode fabrication; an example of this is the fully polymeric, transient SEA developed by Fanelli et al. (2022). Since this device was designed to degrade *in vivo*, the water solubility of the material does not pose an issue. In chronic applications, this, together with the brittleness of standalone CPs, would compromise long-term stability. CP composites have been proposed as an alternative (Goding et al., 2017; Cuttaz et al., 2019). These can be made by blending CPs with hydrogels or elastomers, thus exploiting the favourable electrochemical properties of the CP whilst granting softer, more stable mechanical properties that also enhance conformability. The use of novel materials or surface treatments will be crucial to enable safe stimulation, especially as devices are miniaturised, vastly expanding the potential future applications of ENIs.

#### 4.1.3 Wireless communication

Wired connections are a typical limitation across NIs, introducing additional failure points and limiting the number and density of electrodes. However, this becomes a critical bottleneck in the context of ENIs. While conventional NIs can route their wires through tissue, in an endovascular device wires must travel through the vessel lumen. Each additional electrode requires an additional wire, further obstructing the vessel lumen. Bypassing this bottleneck will require developing wireless systems that allow sufficient data throughput at the desired depth. These requirements will depend on the end application of the device: the distance between the implant and the external device, the number of recording channels and sampling rate, and the stimulation parameters employed (Kim and Ho, 2022).

Wireless communication to endovascular devices has been investigated at length for self-reporting stents (Takahata et al., 2006; Chen et al., 2018; Herbert et al., 2022; Oyunbaatar et al., 2023). Most of these systems employ inductive coupling, taking advantage of the shape of a stent, which makes it well-suited to act as a receiving coil. For this, longer and wider receiving coils would be more effective as antennas. Wireless power transmission to a stent has been achieved at depths of a few centimetres using near-field power transfer, whilst far-field transfer methods have been used to achieve signalling over ranges greater than 50 cm (Bateman et al., 2025). Most of this work has focused on supplying power to read sensor measurements. More recently, this has also been explored for endovascular ECoG (Xu et al., 2026). Through a combination of inductive and capacitive coupling, this work achieved sufficient power transfer to power recording from an SEA. Although this method may not be suitable for powering devices in deeper brain regions, it proves the feasibility of wireless recording from ENIs. On the other hand, power requirements for electrical stimulation are significantly higher (Kim and Ho, 2022). A magnetoelectric implant made employing piezoelectric materials has enabled endovascular stimulation of peripheral nerves with 3 V monophasic pulses by delivering 4 mW power at a transmission depth of 4 cm (Chen et al., 2022). For CNS applications, the skull results in further signal attenuation, possibly reducing the operating range. Additionally, in this case the 95 mm^3^ device was too large for implantation within a blood vessel. Thus, further miniaturisation would be required prior to endovascular implantation.

#### 4.1.4 Vascular safety and long-term stability

As ENIs move towards chronic applications and novel devices, ensuring vascular safety and long-term stability will be necessary prior to their widespread clinical translation. Endovascular devices should be assessed for their impact on vasculature, haemodynamics, and blood components.

Interactions with blood vessel walls are particularly important in chronic applications. Damage to the endothelial lining can expose prothrombotic factors and lead to thrombosis (Marei et al., 2022). Chronic mechanical pressure on the vessel wall can trigger excessive proliferation of smooth muscle cells, leading to neointimal hyperplasia (Cornelissen and Vogt, 2018). Stent design can be optimised to reduce risks, with smooth, thin struts generally minimising neointimal hyperplasia (Hoffmann et al., 2001; König et al., 2002). Corrugated ring stent designs have also been found to decrease arterial injury and neointimal hyperplasia in comparison to slotted tube stent designs (Hoffmann et al., 2001). More flexible devices lead to a reduced neointimal reaction in comparison to rigid stents, whilst enhancing adaptability to tapered or curved vessels (Fontaine et al., 1994). Additionally, stents must be selected to closely match the diameter of the implantation vessel, as oversized stents greatly increase vascular damage (Chen et al., 2013; Wen et al., 2025). To further reduce the risk of neointimal hyperplasia, most stents used in cardiovascular applications incorporate drug-eluting abilities to release antiproliferative agents. Similar technologies could be employed for ENIs. For example, Diao et al. (2025) have investigated the incorporation of drugs onto a degradable silk fibroin stent scaffold for sustained release from an SEA. Implant health could also be monitored by incorporating pressure sensors or monitoring impedance to assess tissue growth, as previously shown in smart stents (Shedden et al., 2010; Chen et al., 2018). ENIs may also affect vessel walls via electrical stimulation. While long-term data on endovascular stimulation remains limited, further investigation into its effects on electroactive smooth muscle cells may be of interest.

Endovascular devices must be designed to minimise vessel occlusion and preserve normal haemodynamics. Regions of static or turbulent flow can result in thrombus formation, and this risk is heightened in arteries with high blood velocities (Sakariassen et al., 2015). Minimising device footprint and developing thin, low-profile devices that reduce lumen occlusion is key to mitigate these issues. Computational modelling of blood flow is commonly used to aid design in stent development (Qi et al., 2024). As connecting leads and wires in ENIs occlude the vessel lumen, number and thickness of wires should be minimised where possible. This further incentivises the integration of wireless power transfer to ENIs.

Foreign materials can affect blood components by inducing haemolysis or thrombogenesis. Thus, novel devices require thorough haemocompatibility testing. Hydrophilic, smooth, low energy surfaces tend to be better suited for blood contacting applications (Kuchinka et al., 2021). Still, due to the high risk of thrombosis, patients usually require 6 to 12 months of antithrombotic therapy after stent implantation (Magnani and Valgimigli, 2016). This introduces an additional risk of haemorrhage. In the case of ENIs, human trials of the Stentrode™ report the use of dual antiplatelet therapy from 2 weeks pre-implantation up to 3 months post-implantation, followed by aspirin for at least a year (Mitchell et al., 2023).

Studies on the chronic vascular response to ENIs are limited and constrained to SEAs due to the lack of anchoring of catheter-based devices. Most of the research in this aspect has been done on the Stentrode™, which was found to become fully endothelialised within 2 weeks post-implantation (Opie et al., 2017). Conversely, others found no indication of neointimal growth up to 28 days after the implantation of a polymeric micro endovascular probe (Zhang et al., 2023). This disparity is likely to be a result of the use polymeric materials, which would also result in lower radial force, or of implantation in a smaller blood vessel with wall thickness of only 10 to 20 μm. This highlights how tissue responses vary significantly across devices and thus must be characterised in detail. Although the Stentrode™ human trials have been ongoing for over 5 years with no major complications reported, no further studies have been published past the one-year timepoint. Longer-term data will also be beneficial to analyse the chronic response to ENIs accurately. This could be estimated using data from stent implantation in the cardiovascular field. The most common long-term complication of stenting in arteries is in-stent restenosis, with an incidence of 17%–41% for bare metal stents and < 10 % for drug eluting stents (Buccheri et al., 2016). Although most of these events occur on the first year upon stent implantation, an adverse event rate of 2% per year remains up to at least 5 years (Cutlip et al., 2004; Madhavan et al., 2020). On the other hand, late stent thrombosis has an incidence of approximately 0.4% to 0.6% per year (Serruys and Daemen, 2007). Cerebral sinuses, which are usual targets of ENI implantation, are less commonly stented for cardiovascular applications. Yet, cerebral venous stenting is a common treatment for idiopathic intracranial hypertension. Although both late stent thrombosis and restenosis remain existing risks, the risk of in stent restenosis due to neointimal growth is significantly lower in venous sinuses (Al-Mufti et al., 2019). It should be noted that these complication rates are obtained from implantation in diseased vasculature. Most cases of in-stent restenosis are likely to be due to the progression of the underlying condition that calls for stent implantation in the first instance. ENIs are implanted in healthy vessels, which have not been previously investigated under stenting, but are likely to behave differently.

The potential risks of ENIs are further aggravated by the inability to retrieve or explant stent-based devices. For cardiovascular stents, this usually results in implantation of additional stents upon device failure. A potential solution could be the use of transient devices, such as the SEA developed by Fanelli et al. (2022). These bypass the issue of explantation by degrading *in vivo* but therefore have a limited lifespan. Ultimately, this is an inherent barrier to translation than chronic ENIs will have to overcome by demonstrating a level of safety and performance that outweighs their risks.

### 4.2 Future opportunities

Currently, ENIs have mostly been employed for intravascular EEG recording for BCI applications or detection and localisation of epileptic seizures. Ultimately, achieving intracranial recording without a craniotomy represents a major advantage, and the upcoming possibility of deploying multiple ENIs would enable whole-brain EEG monitoring with unprecedented resolution (Masuda et al., 2025). Possible future applications include neuromodulation for neurological disorders potentially treatable via traditional deep brain stimulation, including Parkinson’s disease, drug resistant epilepsy, treatment resistant depression, and obsessive-compulsive disorder (Neudorfer et al., 2020; Opie and O’Brien, 2022). The specific conditions that may benefit from this approach are constrained by the vascular locations accessible for ENI implantation. Theoretically, any deep brain region would be a possible target if the device is small enough. Pathways to many of these regions are tortuous and highly variable between subjects, making the procedure technically challenging. Readily accessible vascular targets relevant to the treatment of neurological disorders have been thoroughly reviewed (Teplitzky et al., 2014; Rajah et al., 2018; Neudorfer et al., 2020; Brannigan et al., 2024; Kashyap et al., 2025). Implantation in submillimetric vasculature is likely to call for the development of improved delivery techniques to mitigate the complexity of the procedures. On the other hand, implantation of the device in the SSS, which is on the surface of the brain, is unlikely to exploit the benefits of ENIs in comparison to surface ECoG devices. Differences in performance would be enhanced when targeting deep brain structures, as ENIs could be implanted in nearby vessels closer to the tissue. Thus, the greatest impact of this technology may come for conditions such as treatment resistant depression, obsessive-compulsive disorder, or addiction, which benefit from neuromodulation, but where the risk-to-benefit trade-off does not justify the use of invasive deep brain stimulation.

The spatial resolution of a neural recording is governed by the electrode size and the distance between electrodes and target neurons. For endovascular devices, this distance is limited by the thickness of the blood vessel wall. This inherently introduces an additional separation to the target in comparison to penetrating electrode arrays, and limits the target region to tissue adjacent to the vessel trajectory. Penetrating arrays may allow denser coverage of arbitrarily shaped areas. However, it is worth considering that arrays in direct contact with neural tissue often trigger the formation of a high-impedance glial scar (Campbell and Wu, 2018). This acts as a barrier between the electrode and target tissue, with chronic implantation results showing a 63% decrease in neuron density in the 400 μm × 400 μm area surrounding electrode shanks in penetrating Utah arrays (Patel et al., 2023). Ultimately, this introduces an application-dependent trade-off between ENIs, which are implanted in a minimally invasive manner but are separated from neural tissue due to the vessel wall, and penetrating or surface electrode arrays, which can sample a greater area but may also induce separation from the target due to glial scar formation. It has been shown that different electrode contacts on the Stentrode™, which are estimated to be approximately 260 – 680 μm away from neural tissue, record or stimulate distinct neuron populations (John et al., 2018; Opie et al., 2018). Upon implantation in a blood vessel with wall thickness of 10 – 20 μm, a micro-endovascular probe featuring 80 μm diameter electrodes was able to record single-unit spiking activity (Zhang et al., 2023). This proves that single neuron resolution is achievable endovascularly, albeit only from small vessels requiring miniaturised devices and specialised angiographic implantation procedures. Improvements to ENIs that enable placement in smaller vessels with thinner walls may eventuate in comparable electrode-neuron distances with penetrating interfaces while mitigating loss of signal quality typically associated with the foreign body response.

Whilst most research in the field of ENIs has focused on the CNS, peripheral nerve stimulation could also benefit from this technology. The proximity of nerves and vasculature in the neurovascular bundle can make the use of conventional nerve cuff electrodes challenging but becomes an advantage in terms of endovascular stimulation. This could have many implications, since peripheral nerve stimulation is being investigated for the treatment of chronic pain conditions, urinary incontinence or phantom limb pain (Ganji-Angirekula and Javed, 2025). Interestingly, most findings on endovascular stimulation of peripheral nerves originate from research in the cardiovascular field and are published in cardiology journals. This has resulted in a gap across which knowledge is not readily transferred. Endovascular autonomic nerve stimulation has shown potential as a treatment for central sleep apnoea, transient hypotensive episodes, and increased ventricular rate in atrial fibrillation (Schauerte et al., 1999; Ponikowski et al., 2011; Madhavan et al., 2014; Naksuk et al., 2016). Limitations included the high amplitude thresholds required to achieve a significant effect, which induced side effects such as muscle contraction and pain due to off-target nerve activation. This could partly be attributed to implantation in large blood vessels, such as the superior vena cava, with thicker walls. This increases the distance between the electrodes and target axons, increasing the current required for activation. Implantation in blood vessels with thinner walls may decrease the stimulation amplitude thresholds. Additionally, more selective stimulation could be achieved by stimulating the nerve at a more distal point, after further branching. This was shown by Schauerte et al. (2001), who reported fewer side effects when delivering stimulation from the coronary sinus in comparison to the superior vena cava. The atrioventricular node is mostly controlled by cardiac parasympathetic nerve fibres that are more abundant in the coronary sinus than in the superior vena cava (Schauerte et al., 2001). An additional consideration arises from the choice between arterial and venous implantation. Although navigation in the venous space can be challenging due to the presence of valves or arachnoid granulations, arteries generally exhibit thicker walls, increasing the distance between the device and the target tissue (Tucker et al., 2023). Additionally, stimulation in arteries could result in contraction of the more abundant smooth muscle cells, leading to vasoconstriction. Future research efforts should focus on localising optimal implantation locations for different PNS applications to reduce stimulation thresholds and side effects.

Another key takeaway of the work on autonomic peripheral nerve stimulation is the importance of achieving stable contact with the vessel wall for efficient stimulation. Many have highlighted the potential of a chronic, stable endovascular implant as a novel treatment for central sleep apnoea or hypotensive episodes (Ponikowski et al., 2011; Madhavan et al., 2014). To this end, spiral leads or lasso catheters were developed for improved wall contact (Hasdemir et al., 2003; Nabutovsky et al., 2007; Naksuk et al., 2016). However, most of the work focused on demonstrating feasibility by employing existing devices in acute settings only. Although this lack of stability presents an issue for the development of chronic treatments, the use of approved devices has also enabled clinical studies in patients, which are very limited in ENIs for neural applications. Cardiovascular applications could also benefit from neural stimulation paradigms regarding safe stimulation parameters, tissue damage thresholds, and selective stimulation techniques. Collaboration between professionals in the fields of interventional cardiology and endovascular neural interfacing could accelerate development in both areas, with approved devices potentially being used to investigate what is feasible and effective with safe stimulation parameters. The contact and stability issues reported when using catheter-based electrodes can then be addressed by employing SEAs, whilst assessing device performance to inform further development.

The form factor of SEAs inherently makes them susceptible to the same risks as those associated with stents and as such, they are likely to also benefit from the technologies developed for self-reporting stents. One of the main risks of ENIs is the formation of thrombus on the device surface. The integration of pressure sensors onto SEAs would allow early detection and treatment of thrombosis (Takahata et al., 2006; Chen et al., 2018; Herbert et al., 2022; Oyunbaatar et al., 2023; Bateman et al., 2025). Additional functionalities could also be used to inform the treatment of specific conditions, forming closed-loop systems that integrate both sensing and stimulating abilities. Intracranial pressure monitoring through pressure sensors could provide additional information for the treatment of epilepsy, where pressure is elevated transiently during seizures, or stroke, where patients often exhibit pressure instability (Lee et al., 2025; Sič et al., 2025; Spoor et al., 2025). Together with neural recording abilities, this would enable continuous monitoring of a patient’s condition towards a more targeted, personalised treatment with fewer side effects, whilst significantly decreasing the power requirements of the device. Although research on active stents dates back to 2006, they are yet to be translated past *ex vivo* studies (Takahata et al., 2006). The benefits of smart stents may not justify the additional cost and uncertainty in comparison to more established traditional stents. However, translation of this technology might be accelerated by incorporating active stent functionalities to SEAs, which already have electronics integrated onto them, and where there is not a well-established safer alternative. Explantability of the device is also an important consideration. Currently, stent-based devices are permanent, and a removable device may facilitate translation. Although ENIs have been shown to remain stable up to 1 year post-implantation, additional data and longer-term experiments will be necessary to guarantee long-term safety.

Inspired by the minimally invasive delivery method used for ENIs, recent research has explored using catheters to deliver devices beyond the vascular system. Advancements include an ultraflexible implantable neural electrode (uFINE) that is routed to the target location endovascularly and can then pierce through the vasculature to sit in direct contact with neural tissue (Wang et al., 2024). Others have investigated the possibility of guiding a catheter through the ventricular and subarachnoid space; such devices have been labelled endocisternal neural interfaces (Chen et al., 2024). Although these share some similarities with endovascular devices, because they interface with distinct tissue types, their design requirements and considerations differ substantially. Although these share some similarities with endovascular devices, because they interface with distinct tissue types, their design requirements and considerations differ substantially.

The development of the Stentrode™ has triggered a paradigm shift for the use of ENIs in BCI applications. Deep brain stimulation, peripheral nerve interfacing, closed loop neuromodulation and whole-brain intracranial recording could become minimally invasive, accessible techniques through the implementation of ENIs. Although scientific understanding of ENIs is growing rapidly, the full capabilities of this technology are yet to be fully assessed. Combining the learnings from endovascular approaches in cardiac and neural applications will accelerate the translation of these technologies and define new frontiers in neural recording and stimulation.

## 5 Conclusion

Endovascular procedures have revolutionised the treatment of cardiovascular conditions, and novel fabrication techniques have allowed the development of complex electroactive devices with enhanced capabilities. ENIs apply this approach to the nervous system, offering a minimally invasive and potentially safer alternative to traditional NIs. The endovascular route has been shown to record neural activity up to single-cell resolution and to safely stimulate peripheral nerves, whilst SEAs offer a stable platform for this. To accelerate advancements in this area, future work should incorporate findings from the cardiovascular field, especially regarding endovascular stimulation of autonomic peripheral nerves and the development of active stents with additional functionalities. Several challenges remain, including device miniaturisation, choice of materials, incorporation of wireless power transfer capabilities and ensuring long-term vascular safety. Addressing these could revolutionise the treatment of neurological disorders, improving the quality of life of millions worldwide.
